# Detecting conservation benefits of marine reserves on remote reefs of the northern GBR

**DOI:** 10.1371/journal.pone.0186146

**Published:** 2017-11-08

**Authors:** Carolina Castro-Sanguino, Yves-Marie Bozec, Alexandra Dempsey, Badi R. Samaniego, Katie Lubarsky, Stefan Andrews, Valeriya Komyakova, Juan Carlos Ortiz, William D. Robbins, Philip G. Renaud, Peter J. Mumby

**Affiliations:** 1 Marine Spatial Ecology Lab, School of Biological Sciences, The University of Queensland, Brisbane, Queensland, Australia; 2 ARC Centre of Excellence for Coral Reef Studies, Brisbane, Queensland, Australia; 3 Khaled bin Sultan Living Oceans Foundation, Annapolis, United States of America; 4 School of Environmental Science and Management, University of the Philippines, Los Baños, Philippines; 5 State of Hawai'i Division of Aquatic Resources, Honolulu, United States of America; 6 School of Biological Sciences, The University of Western Australia, Crawley, Perth, Western Australia, Australia; 7 School of BioSciences, University of Melbourne, Parkville, Victoria, Australia; 8 Wildlife Marine, Perth, Western Australia, Australia; 9 Department of Environment and Agriculture, Curtin University, Perth, Western Australia, Australia; 10 School of Life Sciences, University of Technology Sydney, Sydney, New South Wales, Australia; Department of Agriculture and Water Resources, AUSTRALIA

## Abstract

The Great Barrier Reef Marine Park (GBRMP) is the largest network of marine reserves in the world, yet little is known of the efficacy of no-fishing zones in the relatively lightly-exploited remote parts of the system (i.e., northern regions). Here, we find that the detection of reserve effects is challenging and that heterogeneity in benthic habitat composition, specifically branching coral cover, is one of the strongest driving forces of fish assemblages. As expected, the biomass of targeted fish species was generally greater (up to 5-fold) in no-take zones than in fished zones, but we found no differences between the two forms of no-take zone: ‘no-take’ versus ‘no-entry’. Strong effects of zoning were detected in the remote Far-North inshore reefs and more central outer reefs, but surprisingly fishing effects were absent in the less remote southern locations. Moreover, the biomass of highly targeted species was nearly 2-fold greater in fished areas of the Far-North than in any reserve (no-take or no-entry) further south. Despite high spatial variability in fish biomass, our results suggest that fishing pressure is greater in southern areas and that poaching within reserves may be common. Our results also suggest that fishers ‘fish the line’ as stock sizes in exploited areas decreased near larger no-take zones. Interestingly, an analysis of zoning effects on small, non-targeted fishes appeared to suggest a top-down effect from mesopredators, but was instead explained by variability in benthic composition. Thus, we demonstrate the importance of including appropriate covariates when testing for evidence of trophic cascades and reserve successes or failures.

## Introduction

Fishing on coral reefs has taken place for millennia, but ecological impacts have increased with a global intensification of fish stock exploitation [1, 2] and habitat degradation [3–5]. To counteract ecosystem impacts of fishing on reefs, no-take marine reserves have been widely implemented as a management strategy for preventing overfishing, by facilitating the recovery of fish stocks and conserving biodiversity [6–9]. However, the benefits of marine reserves for fisheries and the ecological impacts of marine reserves on restoring fish populations on nearby coral reefs and for benefiting fisheries remains controversial (e.g. [10–14]).

Detecting the primary benefits of no-take marine reserves, such as the protection and restoration of overfished species, requires appropriate context due to the numerous factors influencing the magnitude and direction of effects [15–18]. For example, while no-take marine reserves can allow the recovery in density, size and biomass of targeted species in both tropical and temperate systems [8, 9, 19–22], positive effects on fish density can also be influenced by the size and age of marine reserves [17, 23–25]. In addition, recovery rates differ among species with different life-history characteristics and fishing vulnerability [26, 27], and among size-classes within fish families [8]. For example in Kenya, overall parrotfish density recovered within 10 years of protection whereas acanthurids took more than 30 years [8]. In contrast, dramatic increases in the density of the coral trout (*Plectropomus* spp., Serranidae) were observed only two years after the implementation of no-take reserves on the Great Barrier Reef (GBR; [28]).

The positive impacts of marine reserves on fish populations can potentially be confounded by habitat differences because of the high dependence of reef fish on habitat structure [29–32]. Yet patterns of habitat structure might reflect the impact of exogenous drivers unassociated with reserves and may serve to distort putative reserve benefits. While marine reserves can mitigate habitat damage associated with destructive fishing practices (e.g. dynamite and trawl fishing), they offer little or no protection from anthropogenic stressors such as pollution or nutrients, and climate-driven disturbances such as coral bleaching and cyclones [33–35]. Consequently, differences in habitat quality among reefs may obscure the impact of protection from fishing on fish biomass. Similarly, while the establishment of marine reserves can also affect top-down interactions between predators and prey (e.g. [36–41], but see [42]), the impact of marine reserves in restoring trophic interactions can be difficult to detect if habitat quality varies across reserve boundaries (e.g. [31, 43]). However, relatively few studies assessing the performance of marine reserves have accounted for the effect of habitat on the spatial variability of fish species [19, 41, 44–48].

A typical metric of reserve effectiveness is the contrast in fish biomass between the reserve and comparable fished areas [18]. However, the magnitude of this difference is highly sensitive to the intensity of fishing, and small effect sizes might be expected in relatively lightly-exploited systems. Relatively few reserve studies examine healthy environments, perhaps because of their general scarcity, but the far-northern regions of Australia’s Great Barrier Reef Park (GBRMP) are a prime example of lightly-exploited reefs. The GBRMP, declared in 1975, comprises the world’s largest system of coral reefs stretching 2,300 km along Australia’s north-eastern coastline and extending up to 300 km offshore [49]. The GBRMP is managed by the Great Barrier Reef Marine Park Authority (GBRMPA) through a spatial zoning plan, each zone providing for increasing levels of protection and various types of resource use [49]. While some parts of the GBRMP are open to both commercial and recreational fisheries (‘Blue’ zones), some areas are set aside as ‘Green’ zones that are putatively free from fishing and collecting but allow boating, snorkeling and diving activities (no-take zones). Other no-take areas are also inaccessible for any human activity (‘Pink’ or ‘no-entry’ zones) [49]. Both recreational and commercial fisheries on the GBR are fairly species-specific, focusing primarily on higher trophic levels (i.e. piscivorous reef fishes) using hook and line, which has little direct impact on habitat structure [50]. While fishing intensity on coral reef areas within the GBRMP is relatively low at a regional scale, the commercial fishery has undergone notable increases in effort (by 40%) and catch (by 50%) since 1995 [51]. However, conservation management strategies for a better representation of biodiversity within no-take areas resulted in the modification of the zoning plan in 2004 increasing the reef area closed to line fishing from less than 5% to nearly 34% [51]. In the northernmost section of the GBR (the Far-Northern section) situated east of the Cape York Peninsula, the zoning plan, which has been in operation since 1986, allows for commercial and recreational fishing activities in nearly 84% of the area (i.e. Blue zone), while 13% of the area is a no-take Green zone and less than 1% is a no-entry Pink zone [49].

Recreational fishing in the Far-Northern Section of the GBR is mainly seasonal (from April to October) and is naturally limited spatially and in intensity because of the remoteness of the area and the low resident population. As a result, most of the recreational fishing effort applies to the nearshore reefs, while the commercial line fishery is currently expanding as operators move away from the more heavily used areas [52]. While coral trout (followed by snappers and the redthroat emperor) represent the most intensively harvested group by recreational fishers in the areas of Cairns and Townsville, the catch of snappers exceeds that of coral trout by almost two-fold further north in the Cooktown area [53]. Limited data are available further north of Cooktown and because of the remoteness and large extent of the Far-Northern Section; enforcement and surveillance presence are also limited [52].

To the best of our knowledge, little is known about the performance of management zoning on the northernmost section of the GBR. However, recent studies have shown that the density, size and biomass of coral trout increase in no-entry and no-take zones of the Cairns/Cooktown Section to Townsville [9, 42], which is located immediately south of the Far-Northern Section of the GBR. Interestingly, the densities of sharks and some other targeted species appear to be as great in no-entry zones than no-take zones, which has been attributed to illegal fishing in no-take zones [21, 54, 55]. Another possible mechanism for the higher abundance of some fishes in no-entry zones is the reduction of negative effects associated with shipping noise. For example, recent studies have found that noise-generating human activities reduce the ability of fish to detect predators [56].

Because reefs in the far-north are generally less exposed to cyclones, crown-of-thorns starfish (COTS) outbreaks, and eutrophication compared to the rest of the GBR [33, 34, 57], this section of the GBR provides an opportunity to assess the direct effects of management (i.e. protection from fishing) on the exploited fish stocks in relatively undisturbed benthic habitats. It also offers the opportunity to assess abundances in the face of limited exploitation compared with the rest of the GBR. This study evaluates the impact of management zoning on the coral reef fish assemblages of the northernmost GBR (i.e. Far-North Section and north portion of the Cairns/Cooktown Section). Specifically, we assess the effect of a gradient of protection from fishing (no-entry, no-take, and fished zones) on fish assemblages following a latitudinal gradient and account for reef location on the continental shelf (inshore, mid-shelf and outer reefs), as well as for the impact of habitat effects via the benthos. Because we expect lower fishing intensity in more remote areas, we hypothesize that there is a weaker effect of zoning on the biomass of targeted fish species in outer reefs compared to inshore reefs. We also anticipate a stronger effect of zoning in the central and southern sectors compared to northern reefs due to a presumed higher fishing pressure closer to more populated areas (i.e. near to Cooktown). Because fishing pressure in the northernmost GBR is confined to a few highly-targeted species, we also sought evidence of trophic cascades to non-targeted prey species and the benthos. Finally, we performed an exploratory analysis regarding the spatial distribution of fishing effects; specifically, is there evidence that fishers are ‘fishing the line’ and preferentially targeting areas near reserve boundaries [58]?

## Material and methods

### Ethics statement

All research was completed under research permit G14/36867.1 issued on 15 August 2014. The use and entry into zones in the Amalgamated Great Barrier Reef Marine Park Section and the Great Barrier Reef Coast Marine Park were identified and authorized under permit G14/372021 issued on 28 August, 2014 by the GBRMPA.

### Surveyed area and reef sites

**Fig 1:**
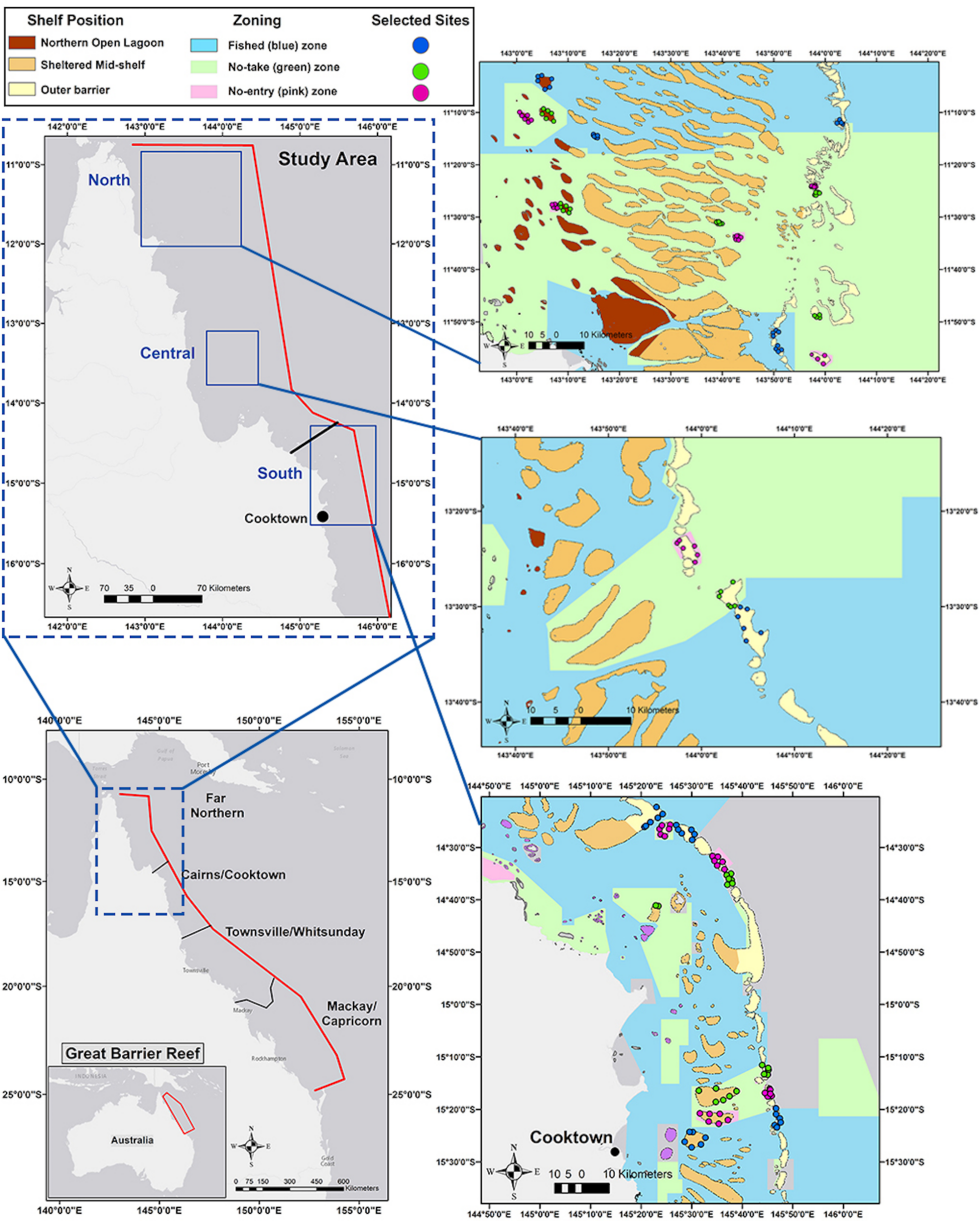
Location of reefs sites surveyed on the northernmost section of the GBR. The legend on the top left indicates the color code to reference each reef by shelf location and management status. Sources: GBRMPA datasets: Great Barrier Reef Features (Version 1.2), Special Management Areas (v1.0), Marine Bioregions of the Great Barrier Reef (Reef) (v2.0). Retrieved from http://www.gbrmpa.gov.au/geoportal. Map created using ArcGIS® software by Esri.

The benthic cover and reef fish assemblage of 31 reefs located along 500 km of the northernmost GBR was assessed by The Khaled bin Sultan Living Oceans Foundation during September 2014 (Fig 1). Surveyed reefs spanned up to 140 km across the continental shelf (inner-, mid- and outer shelf) and each represented one of three management zones officially designated by the GBRMPA: 1) Habitat Protection or Blue Zone which are open to both commercial and recreational fisheries (i.e. fished areas); 2) Marine National Park or Green Zone which allow activities such as diving and boating but prohibit fishing (i.e. no-take areas); and 3) Preservation or Pink Zone which are inaccessible for any human activity (i.e. no-entry areas). All three management zones were surveyed at each shelf position, but not all shelf positions were surveyed along the latitudinal gradient due to logistical constrains (Table 1). Reefs were classified into three sections (sub-regions), hereafter referred as: north (11°01'-11°59'S), central (13°21'-13°34'S) and south (14°21' - 15°27'S). The majority of the surveyed marine reserves (either no-take or no-entry zones) had been implemented for nearly 30 years except for three reefs which were established as no-take zones in 2004 [49]. On each reef, between two and six sites were randomly surveyed, providing a total of 165 sites (Table 1). Therefore, each reef assessment encompassed multiple habitat types (i.e. forereef, back reef and lagoon areas). The exposure of each site was categorized in situ as windward or leeward. Surveys were conducted at a mean depth of 10.0 m ± 0.1 SE per site with transects ranging between 6–15 m depth.

**Table 1:** Characterization of surveyed reef sites.

		GBR Management Sections and sub-regions		
		Far-North		Cooktown
Shelf position	Management zones	north	central	south
	fished	1 (2L-4W)		
Inner lagoon	no-take	2 (7L-5W)		
	no-entry	2 (7L-5W)		
	fished	1 (5L-1W)		1 (3L-3W)
Mid-shelf	no-take	1 (2L-4W)		2 (5L-4W)
	no-entry	1 (2L-4W)		1 (2L-4W)
	fished	3 (7L-5W)	1 (4L-2W)	4 (4L-14W)
Outer	no-take	2 (6L-4W)	1 (5L-1W)	2 (7L-5W)
	no-entry	2 (5L-4W)	1 (3L-3W)	3 (11L-7W)

Spatial location and management status (fished, no-take and no-entry) of surveyed reefs in the northernmost GBR (Far-Northern and Cooktown Sections) across shelf position (inner lagoon, mid-shelf and outer) and sub-regions from north to south. Numbers indicate reefs. Brackets show the number of sites located in leeward (L) and windward (W) sides.

### Benthic cover assessment

Benthic cover was assessed using a point intercept method. The substrate type found every 10 cm along a 10 m transect (i.e. total 100 points/transect) was recorded. Substrate types included: a) live coral; b) algae identified as turfs, sediment-laden turfs, crustose coralline algae (CCA), erect coralline algae or fleshy macroalgae; c) other sessile invertebrates; and d) non-colonisable substrate (e.g. sand). Live coral was identified to genus and main growth form (i.e. branching, tabular *Acropora*, plate (non-*Acropora*), massive, sub-massive, encrusting, foliose or phaceloid). For analyses, all corals were pooled per growth form except tabular *Acropora* which was treated separately due to its dominance and distinctive morphology. In addition, a 10m-long photo transect was conducted at 61 sites (which encompassed 28 reefs) using a 1 m x 1 m quadrat to estimate benthic cover using the Coral Point Count (CPCe) software [59]. A total of 50 points were randomly placed on each quadrat image and the substrate type directly underneath each point was recorded with the same benthic categories as previously described. Due to low replication of the benthic surveys at the level of a site, benthic covers estimated from point intercept and photo transects were treated as replicates after prior analyses revealed no significant effect of the survey method on cover estimates (646 transects in total). Despite this pooling, 40 sites out of 165, had only one sample. Consequently, the effect of benthic structure on fish was analysed at the level of a reef. At this level, one reef (southern outer shelf) was sampled with two (LIT) transects only but all the other reefs had a minimum of seven benthic replicates (S1 Table).

### Fish surveys

Fish counts were conducted using 30 m x 4 m transects. Each transect was conducted by one diver counting fish on both sides of the transect line (within 2 m on each side). A total of four divers performed the surveys throughout the study. On each reef, each replicate site was visited by at least one diver (usually two divers per site). All divers surveyed all habitat types (windward and leeward sites), but not all divers surveyed both fished reefs and reserves within sub-regions due to logistical constraints regarding weather conditions. Therefore, replicate transects ranged between 2–9 per site (712 transects in total). Fish size was estimated to the nearest centimeter using a T square marked in 5 cm increments. Biomass estimates of each species were calculated using published length-weight relationships [60]. A total of 575 species were identified and assigned to a single trophic category: carnivores (i.e. piscivore, piscivore-invertivore, macroinvertivore, microinvertivore), herbivores, planktivores, corallivores, omnivores and others. Carnivores were further categorized as “highly targeted”, “less targeted”, “protected” or “not fished” based on the annual status report of Queensland commercial fisheries [61]. Highly targeted species that dominate the commercial line (and recreational) fisheries include the coral trout of the genus *Plectropomus* (*P*. *areolatus*, *P*. *leopardus*, *P*. *laevis*, *P*. *maculatus*, *P*. *oligocanthus*) and *Variola* (*V*. *albimarginata* and *V*. *louti*) (hereafter referred as coral trout), emperors (*Lethrinus miniatus*, *L*. *nebulosus*, *L*. *erythropterus*), and snappers (*Lutjanus adetti*, *L*. *carponotatus*, *L*. *russelli*, *L*. *sebae*, *and L*. *vitta*). See S2 Table for complete list of carnivores based on importance to fisheries.

### Geographic variables

The location of reefs sites was mapped in ArcGIS 10.4.1 using the reef site coordinates and datasets downloaded from GBRMPA (Fig 1). Geographic attributes such as latitude and distance (km) of reefs to shoreline and distance to the nearest port were estimated to reflect remoteness or accessibility to fishing. Further, the distance (km) to the nearest reserve (either no-take or no-entry) was calculated for each reef where fishing is permitted in order to examine potential ‘fishing the line’ effects whereby fishers preferentially exploit the borders of protected areas. Geographic distances were calculated using the “XY to Line” feature tool and the “Calculate Geometry” equation in ArcGIS 10.4.1. The size of each reef (area in km^2^) was estimated by tracing the reef shoal perimeter using the ‘Measure’ tool in ArcGIS 10.4.1.

### Data analysis

Owing to the large scale of our experimental design, we first explored the effects of management zoning on the benthic community structure, and the fish assemblage structure with respect to latitude (sub-region), shelf position and wave exposure. A Permutational Analysis of Variance (PERMANOVA) based on the Bray-Curtis similarity matrix [62] was performed for the matrix of benthic cover, exploited fish biomass (highly targeted and less targeted species) and non-exploited fish biomass separately. Benthic data were square root transformed and fish biomass was log+1 transformed. Management status (i.e., fished, no-take and no-entry zones), shelf position (i.e., inner, mid-shelf and outer reef), sub-region (north, central and south) and wave exposure (windward and leeward) were included as fixed effects with interactive terms. Sites were included nested within reefs as random effects. We ran PERMANOVA using sums of squares (SS) Type III to account for the unbalanced design [62]. When significant interactions were detected, the interaction term was investigated through *a posteriori* pair-wise comparisons PERMANOVA with adjusted p-values using the Benjamini & Hochberg (BH) procedure. Non-significant terms were removed from the final model. An additional analysis using Similarity Percentage (SIMPER) [63] was performed to determine the benthic categories that contributed the most to the dissimilarity in benthic community structure between fished reefs and reserves. All analyses were performed with the software PRIMER.

Spatial similarity in benthic community and fish assemblage structures among sites was visualized using Principal Coordinates Analysis (PCO) based on the Bray-Curtis similarity matrix [62]. Here, we used site-averaged data in order to correlate benthic and fish variables. Association strength between each PCO axis and vectors of benthic categories and fish species biomass was measured with the Spearman correlation coefficient and visualized by overlaying the vectors onto the PCO plot. The length and angle of the vector shows the strength and type of the association (either positive or negative) with PCO axes [62]. Here, we considered correlations >0.5 to indicate the most influential variables in determining the spatial patterns of similarity among sites.

Initial multivariate analyses revealed 1) strong geographic effects on benthic community and fish assemblages structure; 2) geographic differences in biomass composition between highly targeted and less targeted species; and 3) no significant differences between no-take and no-entry status on the assemblage structure of targeted mesopredators. Therefore, we refined specific geographic locations as combinations of shelf position and sub-regions, and pooled data for the two types of reserves to test separately the effect of zoning (i.e. fished reefs vs reserves) on the total biomass of 1) highly targeted; 2) less targeted; and 3) non-targeted fish using univariate analyses (S3 Table). To allow for adequate replication within each designed geographic location, we grouped reefs as follows: 1) Inshore north reefs (i.e. inner shelf reefs plus one mid-shelf reef); 2) all other mid-shelf reefs (from north to south); 3) outer north reefs; 4) outer central reefs; and 5) outer southern reefs. To account for the concurrent effect of habitat structure on fish biomass we defined new habitat variables using the scores of the surveyed sites along the first two axes of the PCO on benthic community structure. These two new variables are composite descriptors of habitat heterogeneity as they retain most of the variability in benthic cover among sites with the desirable property of being independent (i.e., orthogonal) to each other.

Site-averaged biomasses of highly targeted mesopredators, less targeted mesopredators, and non-targeted species, were investigated separately with linear mixed models (LMM) using the R package *lme4*. Data were log+1 transformed for analyses. Management zones, habitat heterogeneity (defined by the two PCO axes on benthic community structure) and wave exposure were treated as fixed effects, whereas reef, observer, and depth were treated as random effects. Conformity to model assumptions was evaluated by controlling the dispersion and normality of model residuals. We performed a variance components analysis to determine the percentage variance of random effects (i.e. how much variation in biomass is explained by the random effects), and a Restricted Likelihood Ratio Test with the *exactRLRT* function from the *RLRSim* package to test the significance of the random effects in each LMM [64].

Finally, we used linear regression models to test whether the biomass of highly targeted species in fished areas was depleted with greater proximity to reserves (i.e. potential fishing the line). We also included reserve size as a factor in case this mitigated or exacerbated a pattern of fishing adjacent to protected areas. Fish biomass was squared root-transformed in order to meet the assumptions of linear regression analyses.

## Results

### Benthic community structure

**Fig 2:**
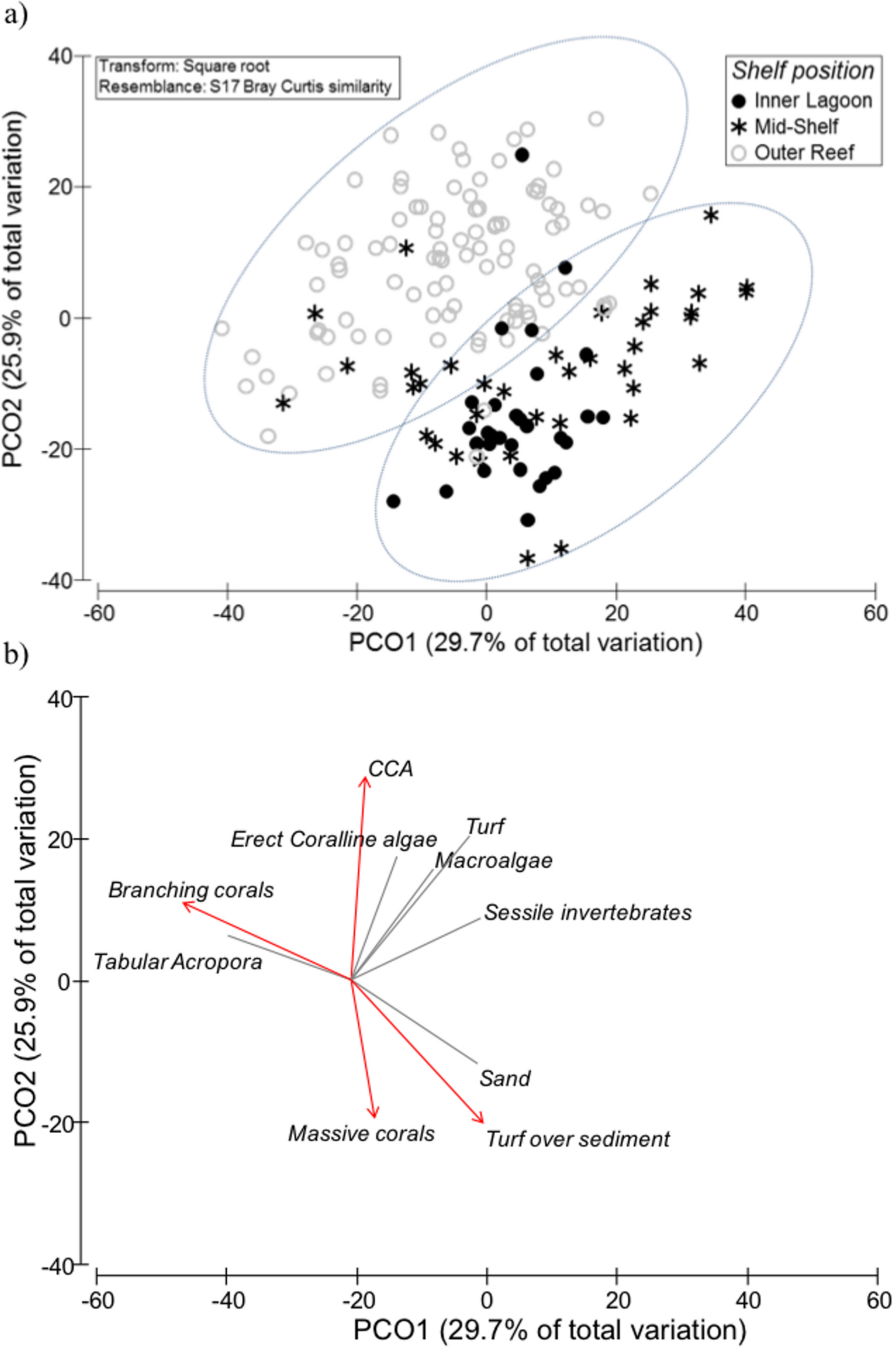
Principle Coordinate Analysis (PCO) of benthic cover. Ordination plots show (a) reef sites along the first two axes based on shelf position, (b) overlay of vectors based on Spearman correlations. Strongest correlations (> 0.5) shown in red. Cover data were square-root transformed for analyses.

**Fig 3:**
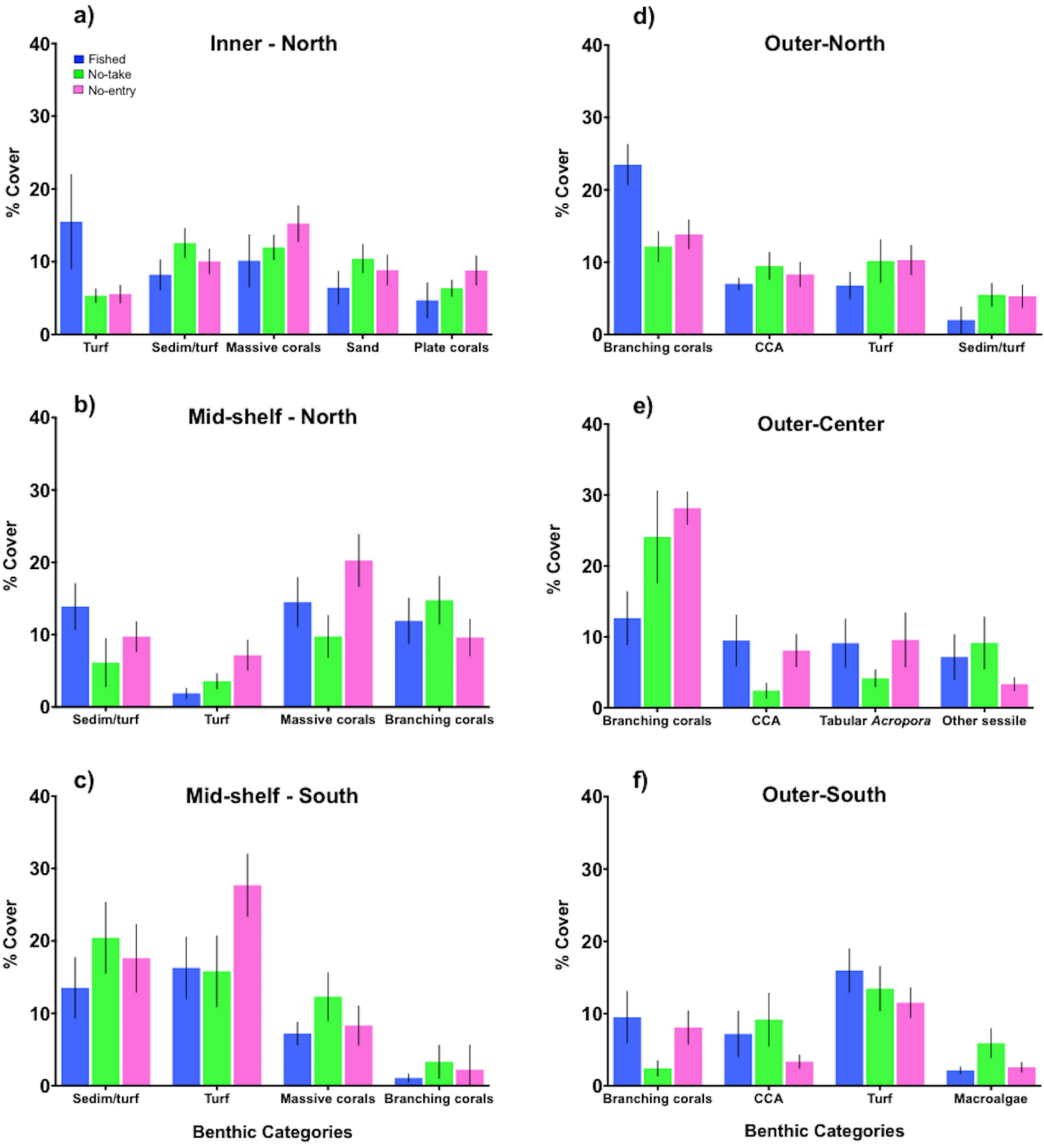
Benthic components that made the greatest contribution to dissimilarity among zones. Mean **%** cover (± SE) of the benthic components identified by SIMPER analyses as major contributors to the dissimilarity in benthic community structure among zones (fished = blue, no-take = green, no-entry = pink) in each sub-region (a-f). Significant differences between reserves (i.e. no-take vs no-entry) were only found in northern mid-shelf reefs and outer-central reefs. Sedim/turf = sediment-laden turfs.

Benthic community structure was strongly associated with position across the continental shelf. The first two PCOs explained nearly 56% of variance and generated two groups of reef sites: (1) inner and mid-shelf reefs vs (2) outer-shelf reefs (Fig 2A). Wave exposure explained little (<1.5%) of the total observed variability and affected only no-take reserves (S4 Table). Those benthic cover vectors with the strongest correlation to the PCO revealed that high cover of branching corals (mainly *Acropora* and *Pocillopora;* S5 Table), tabular *Acropora* corals and CCAs were indicative of outer-shelf reefs as opposed to inner and mid-shelf reefs. Conversely, ‘sediment-laden turfs’ and massive corals were strongly associated with inner and mid-shelf reefs but poorly represented on outer-shelf reefs (Fig 2B). While shelf position was the main predictor of benthic habitat composition, overall, northern reefs had greater total coral cover (44.5 ± 2.2% vs 31.3 ± 2.7%) and lower algal cover than reefs in the south (23.0 ± 1.7% vs 34.3 ± 3.6%). Therefore, significant interactions were detected with sub-region and management zones (PERMANOVA, Table 2). Significant differences between reserves (i.e. no-take vs no-entry) were only found on northern mid-shelf reefs and outer-central reefs (S6 Table). A SIMPER analysis showed that these differences were mainly due to the greater cover of massive corals and CCA in no-entry zones compared to no-take zones (Fig 3). Interactive effects with sub-regions were partly due to branching corals in outer reefs, which achieved higher cover in northern (17.0 ± 1.3%) than southern (10.7 ± 1.1%) areas. Within the northern outer-shelf reefs, the cover of branching corals was higher on fished sites (23.5 ± 3%) compared to protected sites (no-take 12.2 ± 2%; no-entry 13.7 ± 2%). Other noticeable differences among management zones were on northern inner reefs, where the cover of algal turf was greater in fished sites (15.5 ± 6.5%) than reserves (no-take 5.3 ± 1%; no-entry 5.5 ± 1%) (Fig 3).

**Table 2:** PERMANOVA results on benthic community and fish biomass structure.

Response variable	Source	df	MS	Pseudo-F	p-value	% Estimates of Variation
Benthic cover	Shelf position	2	37187	31.8	<0.001	14.3
% cover (sqrt)	Sub-region	2	9236.3	7.9	<0.001	3.7
	Zone*Shelf	4	3697.6	3.2	<0.001	3
	Zone*Sub-region	4	3675.6	3.1	<0.001	3.4
	Residual	624	1169.4			70.4
	Total	645				
Fished species	Shelf position	2	118000	24.1	<0.001	16.89
Biomass (log+1)	Sub-region	2	18142	3.8	<0.001	2.35
	Wave Exposure (WE)	1	10289	2.2	0.01	5.8
	Zone*Sub-region	4	8997	1.9	<0.001	2.1
	Zone*WE	2	7538	1.6	0.04	8.4
	Sites(Reef)	134	5017.7	1.7	<0.001	9.4
	Residual	553	2170.2			53.6
	Total	704				
Non-fished species	Shelf position	2	145000	26.3	<0.001	21.8
Biomass (log+1)	Sub-region	2	21401	4.1	<0.001	2.7
	Sites(Reef)	134	5537.7	2.5	<0.001	18.6
	Residual	556	2229.7			53.9
	Total	704				

Only significant factors explaining more than 2% of total variability are shown.

### Multivariate composition of targeted species

**Fig 4:**
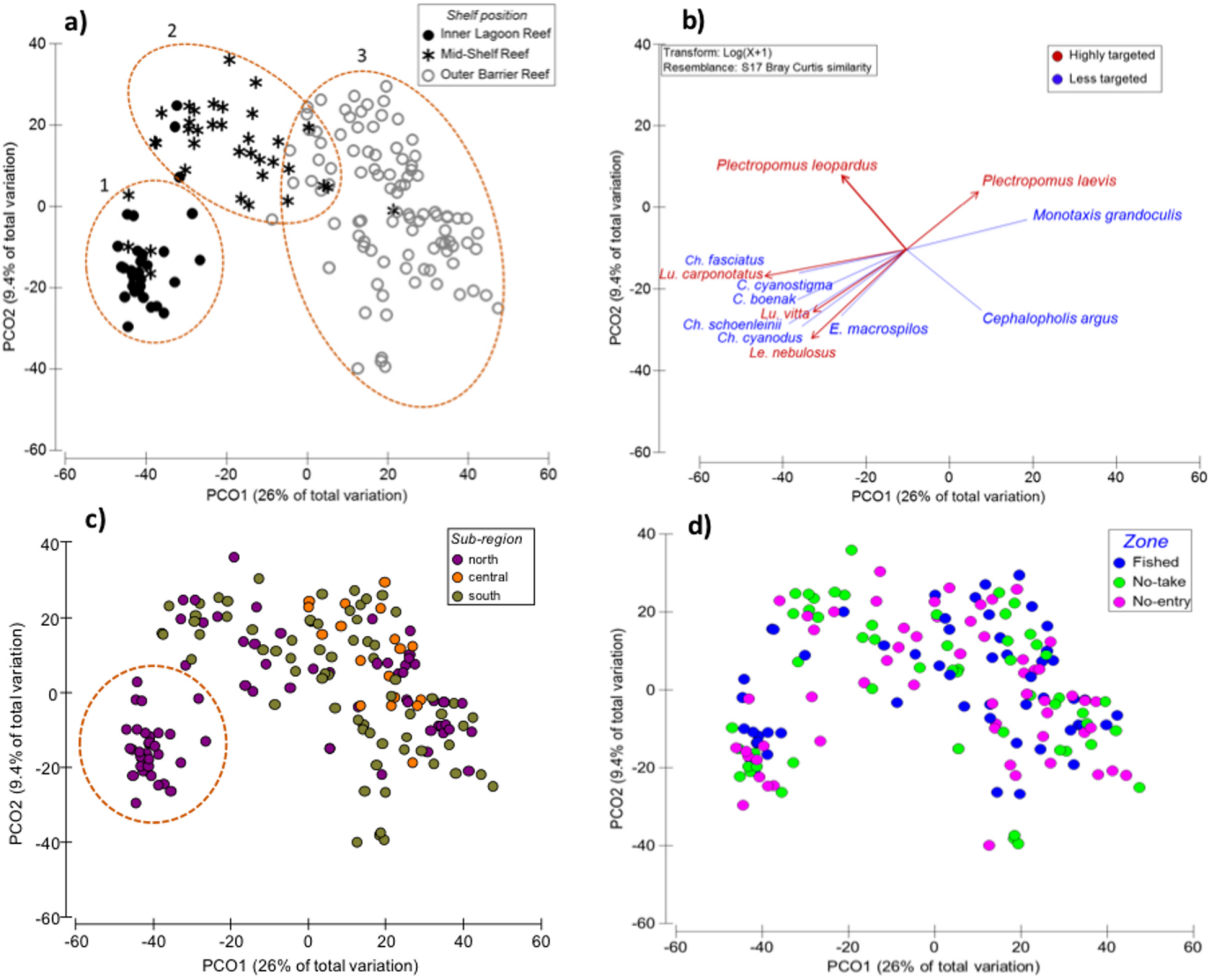
Principle Coordinate Analysis (PCO) of the assemblage structure of targeted carnivores. (a) ordination of reef sites along the first axes (PCO1, PCO2) following shelf position, (b) species of highly targeted (red) and less-targeted (blue) carnivores that show the strongest correlation (Spearman >0.5) to PCO, (c) ordination following sub-regions. Dotted circle denotes inshore reefs (<50 km from shore) and (d) management zones. Biomass was log+1 transformed for analyses.

The biomass composition of all targeted species (i.e. highly and moderately targeted) mirrored the geographic pattern of benthic structure. Fish assemblages were strongly associated with shelf position (Fig 4A and Table 2). In all cases where multiple shelf locations were sampled (i.e., in the north and south), outer-shelf reefs formed a single independent cluster (Fig 4A and 4C). Mid-shelf reefs, which were only sampled in the north and south, exhibited a more complex pattern reflecting distance from shore. The designation of a reef’s shelf position is a relative measure and some northern mid-shelf reefs were geographically closer to inner-shelf reefs than others. As a consequence, those northern mid-shelf reefs that were geographically closer to inner-shelf reefs (<50 km from shore) clustered with inner reefs on the PCO (Fig 4C). Those northern mid-shelf reefs lying farther offshore (typically >90 km from shore) clustered as a single mid-shelf group that also encompassed southern mid-shelf reefs (Fig 3A and 3C). Discrimination of reef sites due to habitat type did not occur (S1 Fig).

Disaggregating fish among highly and less targeted helped characterise the compositional differences in fish community among outer reefs, mid-shelf, and northern mid/inner shelfs (hereafter inshore reefs; Fig 4B). Inshore reefs were characterised by having the greatest biomass of the highly targeted lutjanids (*L*. *carponotatus* and *L*. *vitta*) and the lethrinid *L*. *nebulosus*. This cluster was also characterised by the less-targeted *Cephalopholis* spp. (serranids) and *Choerodon* spp. (labrids). The biomass of the highly targeted common coral trout (*P*. *leopardus*) was highest on mid-shelf reefs, while the lethrinid *Monotaxis grandoculis* was most commonly found on outer-shelf reefs and characterised these environments (Fig 4B). The large *P*. *laevis* was indicative of outer reefs, overlapping with mid-shelf reefs.

Simple partitioning of fish composition by management zones did not occur (Fig 4D). In fact, the effect of management zones varied with shelf position, sub-regions and wave exposure (Table 2, and S6 Table). These patterns are explored in more detail using univariate analyses per fished category (highly targeted and less-targeted).

### Management zoning effects on highly targeted fish (univariate analyses)

**Fig 5:**
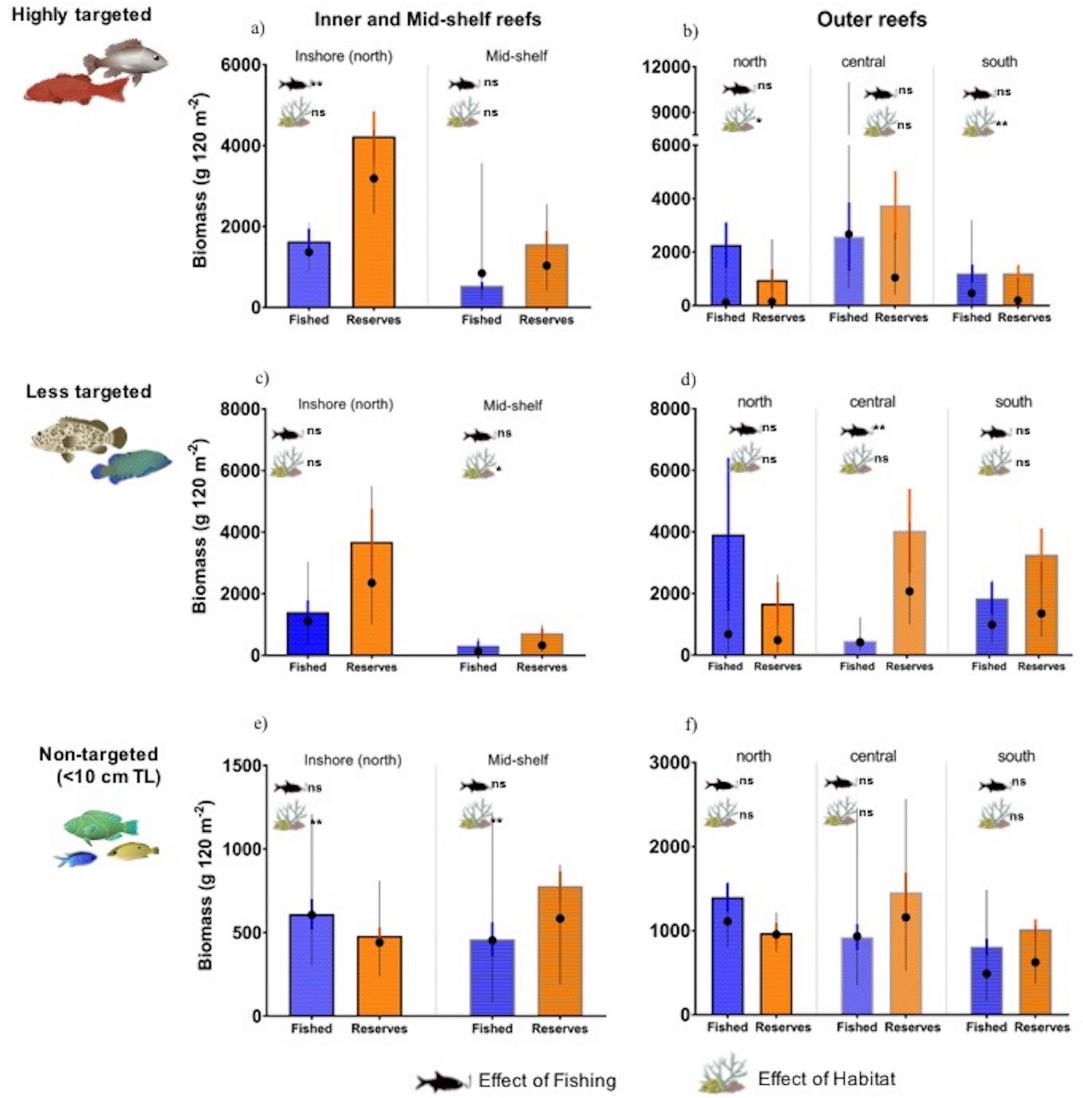
Management zoning effects on fish biomass. Observed biomass (mean ± SE) of highly targeted (a, b), less targeted (c, d) and small (< 10 cm TL) non-targeted species (e, f) in fished reefs (blue bars) and reserves (orange bars) at each geographic location. Black symbols and error bars (mean ± 95% CI) show the biomass predicted by the linear mixed model due to the individual effect of management status (average biomass plus estimated effect of management). Statistical differences associated with either management and/or habitat (i.e. benthos or wave exposure) are denoted: significant (**≤0.05), marginal (* = 0.06), non-significant (ns).

The biomass of targeted fish was indistinguishable between the two no-take reserve categories (no entry vs no-take) (S6 Table) so they were pooled for comparisons with fished reefs. In inshore reefs, zoning effects were evident in some highly-targeted species, even after accounting for habitat heterogeneity (Table 3 and Fig 5A). Specifically, the biomass of the carnivores *L*. *nebulosus* (85.2 ± 32.7 g 120 m^2^) and *L*. *vitta* (3.4 ± 2.7 g 120 m^2^) was reduced by more than 60% in fished reefs compared to reserves (337.9 ± 80.0 g 120 m^2^ for *L*. *nebulosus* vs 89.9 ± 29.6g 120 m^2^ for *L*. *vitta*) but no differences were detected for the biomass of coral trout (718.3 ± 167.2 g 120 m^2^ in fished reefs vs 716.4 ± 124.8 g 120 m^2^ reserves). No differences in benthic composition were evident between fished reefs and reserves in inshore reefs and exposure did not affect fish biomass. Therefore, habitat did not explain changes in fish biomass among zones corroborating that differences were attributed to zoning only.

**Table 3:** Summary of linear mixed models testing zoning and habitat effects on fish biomass.

Location	Variable	Effect	Estimate	SE	df	t	p-value
Inner–north	Highly targeted	(Intercept)	7.17	0.25	28.00	28.72	<0.0001
	(log+1)	Reserve	0.85	0.29	28.00	2.95	**0.01**
	Less targeted	(Intercept)	7.26	0.61	5.91	11.93	0.00
	(log+1)	Reserve*Windward	1.09	0.58	24.57	1.87	0.07
	Non-targeted	(Intercept)	6.39	0.34	4.28	18.82	<0.0001
	(log+1)	Benthos_PCO2	0.01	0.01	22.49	2.36	**0.03**
MidShelf	Highly targeted	(Intercept)	6.38	0.76	18.54	8.42	<0.0001
north-south	(log+1)	Benthos_PCO2	-0.05	0.03	25.58	-1.95	0.06
	Less targeted	(Intercept)	4.61	0.76	9.03	6.06	<0.0001
	(log+1)	Benthos_PCO1	0.03	0.01	24.04	2.01	0.06
	Non-targeted	(Intercept)	6.26	0.55	3.75	11.43	<0.001
	(log+1)	Benthos_PCO1	-0.02	0.01	21.62	-3.32	**0.003**
		Benthos_PCO2	0.02	0.01	18.87	3.40	**0.003**
Outer–north	Highly targeted	(Intercept)	4.65	1.65	3.42	2.81	0.06
	(log+1)	Benthos_PCO1	-0.05	0.03	23.22	-1.95	0.06
	Non-targeted	(Intercept)	7.06	0.20	7.78	34.55	<0.0001
	(log+1)	Benthos_PCO1	0.01	0.01	24.87	1.73	0.10
Outer–central	Less targeted	(Intercept)	6.36	0.49	9.10	13.03	<0.0001
	(log+1)	Reserve	1.60	0.64	12.99	2.51	**0.03**
		Windward	-1.26	0.67	12.36	-1.88	0.08
Outer south	Highly targeted	(Intercept)	7.03	1.16	7.07	6.08	<0.001
	(log+1)	Windward	-1.77	0.80	40.46	-2.20	**0.03**

Only predictors with p-value ≤0.1 are shown. Significant effects (p≤0.05) are shown in bold. See S7 Table for full results.

In mid-shelf reefs, an apparent two-fold increase in the biomass of highly-targeted species within reserves (Fig 5A) was not attributable to zoning (Table 3). Similar results were obtained when analyzing coral trout separately. The apparent increase in coral trout biomass from 228.2 ± 94.9 g 120 m^2^ in mid-shelf fished reefs to 1274.0 ± 253.9 g 120 m^2^ in reserves (a 5-fold increase mainly attributed to *P*. *leopardus*) was not explained by zoning (Wald test ANOVA, Chisq = 2.42, p = 0.11). Wave exposure did not explain these differences but a weak effect of benthic habitat structure was detected (Table 3). Reefs in the reserves which had back-reef lagoon areas (in 10 out of 26 sites) had a considerably higher cover of branching corals (nearly 15%) compared to fished sites (less than 2%) which all lacked lagoon areas. However, removing lagoon areas did not affect the estimates of branching coral cover within reserves (nearly 15% when lagoon areas are excluded). Consequently, even when similar habitat types were compared (i.e. excluding lagoon areas), no reserve effects were detected on fish biomass (Wald test ANOVA, Chisq = 0.007, p = 0.93). Therefore, benthic habitat cannot be discounted as a co-variate impacting fish biomass.

On northern outer reefs, the apparent lower biomass of highly-targeted species in reserves compared to fished reefs (Fig 5B), was confounded by differences in habitat structure between zones (Table 3) and variability among observers (S8 Table). Lower biomass of coral trout in reserves (924.4 ± 212.0 g 120 m^2^ vs 2189.3 ± 632.0 g 120 m^2^ in fished reefs) was particularly due to *P*. *leopardus*, *P*. *laevis* and *Variola louti* and was not attributed to zoning even when comparing among similar habitat types (i.e. among windward or leeward sites only; Wald test ANOVA, Chisq = 0.74, p = 0.38).

On central and southern outer reefs, the biomass of highly-targeted species did not differ between reserves and fished sites (S6 Table). On central reefs, variability among observers accounted for more than 50% of the variability in fish biomass (S8 Table). Specifically, there was high variability in the biomass of coral trout in central outer reserves (3044.2 ± 1542.7 g 120 m^2^ in reserves vs 2523.6 ± 604.4 g 120 m^2^ in fished reefs). In southern outer reefs, an apparent 5% increase in coral trout biomass inside reserves (1154.0 ± 306.8 g 120 m^2^ vs 1087.6 ± 349 g 120 m^2^ in fished sites), was only explained by differences in wave exposure (Wald test ANOVA, Chisq = 4.54, p = 0.03).

### Management zoning effects on less-targeted fish

In inshore reefs, weak interactive effects of zoning with wave exposure were detected (Wald test ANOVA, Chisq = 3.05, p = 0.06). Wave exposure had contrasting effects on the biomass of less-targeted species and this could have masked any reserve effect (Fig 5C). For example, windward fished sites had nearly 2-fold less biomass than leeward sites (915 ± 374 g 120 m^2^ vs 1751 ± 590 g 120 m^2^), whereas windward reserves had more than 2-fold greater biomass compared to leeward reserves (5475 ± 2150 g 120 m^2^ vs 2062 ± 313 g 120 m^2^). Therefore, we compared among similar habitat types, but only weak reserve effects were detected in inshore-windward reefs (pairwise ANOVA, Chisq = 4.10, p = 0.08). Similarly, zoning effects were not evident in mid-shelf reefs (Fig 5C) probably due to the high variance in surveyor estimates (S8 Table). However, a weak effect of benthic habitat structure was detected (Table 3). When comparing among similar habitat types, a marginal effect of reserves was detected in mid-shelf windward reefs only (pairwise ANOVA, Chisq = 4.60, p = 0.06) while accounting for benthic habitat heterogeneity.

Zoning effects were found on central outer reefs (Table 3). Here, the effect of management protection was significant even after accounting for differences in habitat structure (branching coral cover was 2-fold greater in reserves compared to fished reefs). After factoring out the effect of habitat from the model, a stronger effect of protection from fishing was detected (Wald test ANOVA, Chisq = 3.05, p = 0.008). Differences in fish biomass on outer central reefs were mainly attributed to the macroinvertivore *Monotaxis grandoculis* (Lethrinidae) which had 2-fold greater biomass in reserves (Fig 5D).

### Multivariate composition of non-targeted species

**Fig 6:**
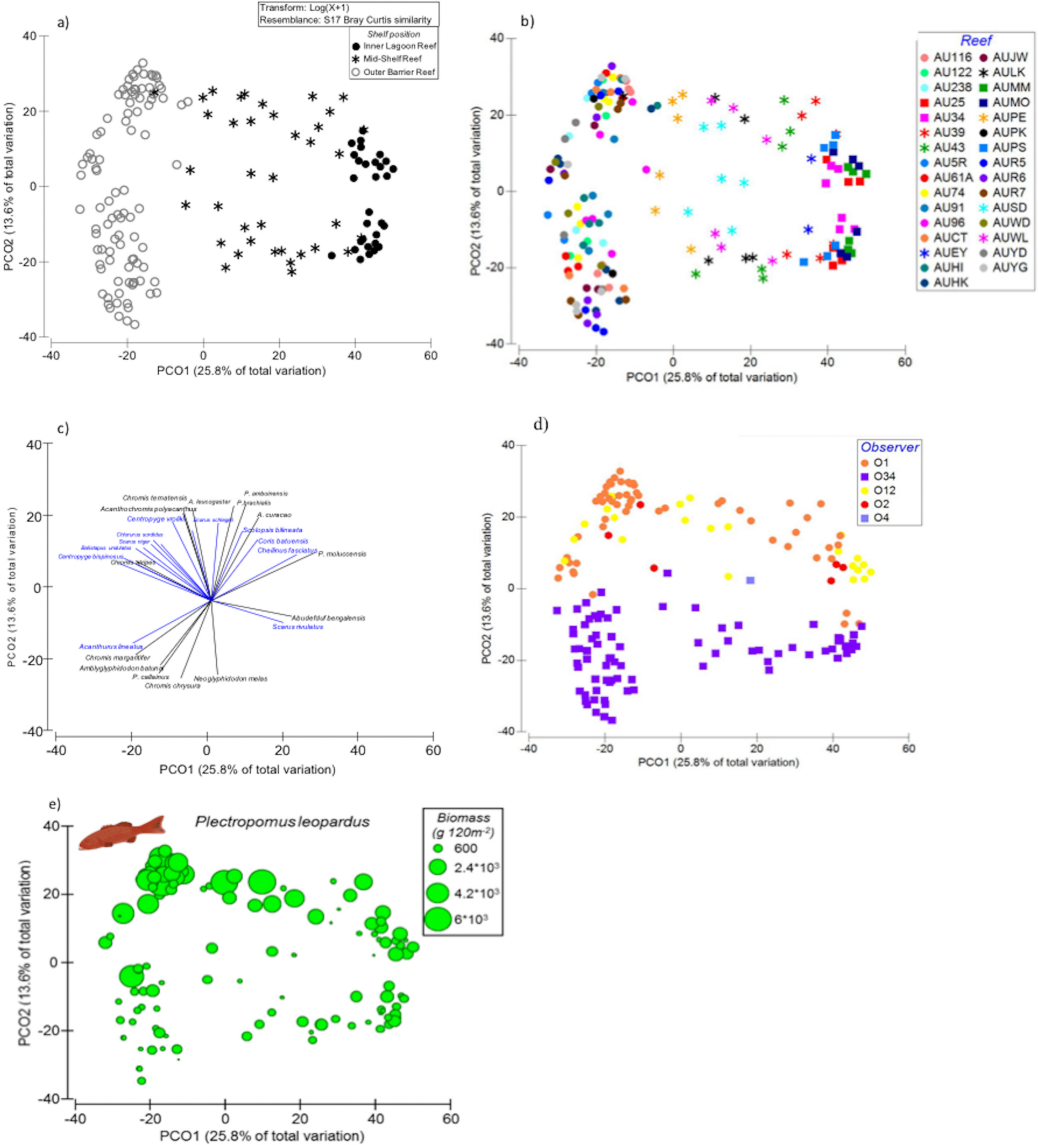
Principle Coordinate Analysis (PCO) of the assemblage structure of non-fished species. Plots show (a) ordination of reef sites along PCO1indicating shelf position, (b) within reef variability along PCO2 (separation of replicate sites of same reef), (c) species with strongest associations (Spearman >0.5) to PCO2: pomacentrids are shown in black, (d) Observer’s identity associated with PCO2 variability, and (e) variation in biomass of *P*. *leopardus* along PCO2. Biomass was log+1 transformed for analyses.

The assemblage structure of non-targeted species was also strongly associated with shelf position which explained nearly 26% of variability (PCO1, Fig 6A and Table 2). Yet within-reef variability was also important, explaining nearly 14% of total composition variability. Replicate sites of the same reef aligned along PCO2 generating two groups (Fig 6B). The first group comprised those species that exhibit a negative relationship with PCO2 (Spearman <-0.5) and included several damselfish species (family Pomacentridae) (Fig 6B). These species, *Chromis chrysura*, *Pomacentrus callainus*, *Amblyglyphidodon batunai* and *Neoglyphidodon melas* form large aggregations of up to 300 individuals and are common on shallow lagoon areas. The second group of species associated positively with PCO2 and represented a more diverse assemblage of species including the planktivorous and omnivorous pomacentrids: *Pomacentrus amboinensis*, *Pomacentrus brachialis*, *Amblyglyphidodon leucogaster*, *Chromis ternatensis*, *Acanthochromis polyacanthus*, *Amblyglyphidodon curacao* followed by other small and medium-size species from the families Labridae, Nemipteridae and Blennidae (Fig 6C). None of the environmental variables–benthic composition, habitat type, wave exposure and depth–showed a strong correlation with PCO2 (S2 Fig). Only a moderately strong correlation (Spearman = 0.5) was found with depth. On the contrary, we found that the divergence of species along PCO2 was strongly associated with observers, suggesting that detectability of small species was highly variable among surveyors (Fig 6D). Interestingly, when the biomass of large predators was overlaid on PCO2, few piscivores were aligned with potential prey. Only *P*. *leopardus*, which was consistently found in most of the reefs sites (in 122 out of 166 sites), exhibited a moderately strong positive correlation (Spearman = 0.53) with PCO2 (Fig 6E).

### Management zoning effects on non-targeted fish (univariate analyses)

In the north, inshore and outer reefs exhibited a similar pattern of prey depletion in reserves but for different underlying causes. While the biomasses of small (< 10 cm) prey in inshore reefs was lower in reserves compared to fished reefs (Fig 5E), this pattern was attributable to differences in habitat structure and observer estimates rather than a reserve effect *per se* (Table 3, S8 Table). In contrast, a similar pattern of depletion for outer reefs (Fig 5F) was not attributable to benthic structure (PCO2, Wald test ANOVA, Chisq = 2.76, p = 0.09). Habitat effects also explained the apparently higher biomass of non-targeted fish in the reserves located in mid-shelf reefs as well, but again, similar patterns on central and southern outer reefs were not attributable to benthic structure (Table 3) and are likely to be explained by variability in observers estimates (S8 Table).

### Evidence for fishing the line

**Fig 7:**
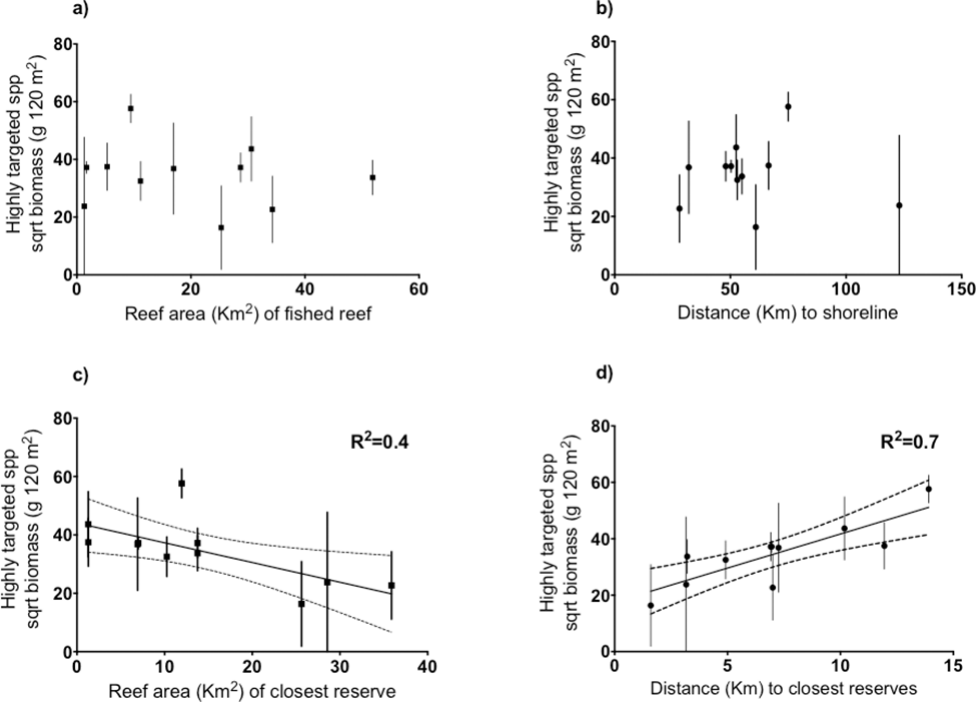
Spatial patterns of biomass of highly targeted species on reefs open to fishing. Fish biomass (mean ± SE) as a function of (a) fished reef size (platform area in km^2^), (b) distance to shore (km), (c) size of the closest reserve (platform area in km^2^), and (d) distance to the closest reserve (either no-take or no-entry zone). Significant correlations (p< 0.05) show best-fit ± 95% confidence intervals. Biomass was sqrt-root transformed for analyses.

The size of fished reefs and their location relative to the shore did not influence fish biomass of highly targeted species (Fig 7A and 7B). On the contrary, biomass was influenced by the proximity of fished sites to the nearest reserve (all located within 14 km; mean 7.0 ± 1.1 km) and reserve size which ranged from 1.3 to 35.8 km^2^. Accounting for habitat heterogeneity and potential surveyor bias, the biomass of highly targeted species decreased with greater proximity to reserves (Fig 7D), particularly near larger reserves (Fig 7C) (ANOVA, t = 2.94, p = 0.005 and ANOVA, t = -2.71, p = 0.008 respectively). Because no such relationships were found for the biomass of less targeted species (ANOVA, t = -1.64, p = 0.13 for distance to reserve and t = -0.25, p = 0.80 for reserve size) or non-targeted species (ANOVA, t = 1.00, p = 0.32 for distance to reserve and t = 1.04, p = 0.33 for reserve size), our results are consistent with a ‘fishing the line’ effect and suggest that the attraction of fishing reserve boundaries is greater for larger reserves.

## Discussion

Because marine reserves provide benefits not only for fisheries, but also for local economies and the environment, assessing the performance of marine reserves is crucial. While the GBRMP is the world’s largest network of marine reserves, to the best of our knowledge, little is known about the performance of management zoning on the remote areas of the northern GBR. Only a few studies have investigated the effect of fisheries management zoning in the area north of Cooktown [42, 55, 65, 66] which corresponds to the southernmost locations of the area studied here. In this relatively undisturbed environment, presumably subjected to moderate to low fishing pressure, we did not find any differences in fish biomass between the two ‘unfished’ zones of no-take and no-entry. However, we did find that fished versus the broader grouping of unfished zones exhibited some impacts on targeted teleosts, but these effects varied in space and were frequently confounded by differences in habitat structure among zones. We found evidence that marine reserves can enhance the biomass of highly-targeted piscivores such as lutjanids and less-targeted carnivores such as the lethrinid *Monotaxis grandoculis* in some of the most remote areas of the GBR, indicating that protection from fishing benefits fish populations even in relatively lightly fished areas. However, a specific analysis on the highly prized coral trout (considering only *Plectropomus* and *Variola* species) demonstrated no significant reserve effects. Importantly, results showed that the spatial distribution of targeted fish outside large reserves may be affected by a “fishing the line” effect [58], i.e., an intensification of fishing along reserve boundaries as an attempt to capture potential spillover from higher fish biomass. Such a shift in fishing effort may affect the detectability of reserve effects at a broad scale.

Positive effects of reserves were detected in inshore reefs where the biomass of highly-targeted lutjanids increased by nearly two-fold compared to fished sites. In contrast, while there was nearly three-fold greater biomass of highly-targeted fish in mid-shelf reserves vs. fished zones, and specifically a five-fold biomass increase for *P*. *leopardus*, these contrasts were not attributed to a zoning effect *per se*, but likely to habitat differences. It is likely that variability in habitat characteristics between zones prevented detection of zoning-related effects. While further sampling is required to corroborate the lack of a reserve effect on mid-shelf reefs, our results on inshore reefs corroborate clear reserve benefits similar to other studies on inshore reefs of the central GBR [9, 67, 68]. While age since implementation has an impact on the effectiveness of marine reserves [24], only three reefs were recently established as no-take zones in this study (10 years of protection). One reef was located inshore where reserves effects on highly-targeted species were clear. The other two were located offshore and a separate analysis of large carnivores revealed that the new reserves appeared to outperform older reserves (~30 years of protection) in the same region.

The magnitude of reserve impacts on targeted species within a given habitat will depend on levels of fishing pressure and compliance (e.g. [67, 69]), which likely decrease from inshore to offshore locations [50]. Reserve effects on offshore GBR reefs have been found to be generally weaker [19] probably due to their greater distance from centres of human population. Here, strong reserve benefits in offshore reefs were only detected for less-targeted species on central-outer reefs. Compared to the intense and non-selective coral-reef fisheries found in many countries like Jamaica[2], Philippines [6], and Kenya [70], reef fisheries on the GBR almost exclusively focus on the highest trophic levels [50]. While it is a highly selective fishery, the intensity of fishing in the northern GBR is expected to be moderate owing to the remoteness of reef areas and the very low density of human population [52].

That reserve effects were found in some of the less-intensively fished parts of the GBR is consistent with observations of lightly-fished systems elsewhere [71] and reinforces the high sensitivity of carnivorous and piscivorous species to fishing [72]. However, discovering that the benefits of protection from fishing were greater on reefs expected to be subjected to the least fishing pressure (here, north inshore reefs) rather than on reefs closer to ports and human settlements (here, southern reefs near Cooktown) was surprising. There are several contributing factors to this apparently paradoxical pattern. First, intense fishing in southern areas may account for lower overall biomass of highly targeted species in that region (i.e., because of greater proximity to ports). Therefore, the relative scarcity of fish in fishable zones may have driven fishers to undertake greater levels of poaching inside southern reserves. This is the case for the central GBR, where poaching levels by recreational fishers are relatively high and reduce reserve benefits [73]. Similarly, relatively low fishing pressure in northern-most areas, which leads to greater stock levels, may reduce incentives to poach and foster greater magnitudes of reserve effects.

An alternative mechanism contributing to variable strengths of putative ‘reserve effects’ is a systematic pattern of greater habitat variation among zones in outer and southern reefs which serves to confound and mask the magnitude of reserve effects. Variation in habitat structure among zones could stem from direct and indirect impacts of fishing on corals that feed back to fish populations. However, direct physical damage to corals because of fishing activities (e.g. [74]) are unlikely because fishing methods in the GBR are essentially non-destructive (hook and line and spearfishing [50]). Similarly, indirect impacts of fishing that favour the development of fleshy algae [75] are unlikely because fishing on the vast majority of the GBR does not target herbivorous fishes (though some trophic effects of mesopredators are feasible). Impacts from stressors such as thermally-induced bleaching, COTS or cyclones that are likely to be more frequent in southern reefs than the far north [34, 57, 76] could contribute to greater habitat variation among zones and partly explain why we did not find consistent differences in the benthic community structure between fished and reserve reefs but rather variation in habitat structure independent of management status. Variation in habitat structure among zones could also result from surveying different habitat types. For example, branching corals were less abundant within reserves on northern outer reefs while on central outer reefs a greater cover of branching corals was found in the reserves compared to fished reefs. Such variations in coral community composition may be explained by differences in wave exposure among reef sites. Because wave exposure also influence distribution of fish with different swimming performances [77], different wave exposures among inshore and mid-shelf reefs may affect the detectability of reserves effects on less-targeted species.

Reserves on the central (albeit northern) outer region were dominated by branching corals (mainly *Acropora* and *Pocillopora*) and it is likely that this, rather than protection status, accounted for the 8-fold increase in biomass of less targeted species in protected zones. High complexity habitats like this frequently have a strong positive effect on fish density and mobile invertebrates [78] and it is likely to impact large predators through facilitation of prey [29–31, 79]. Similarly, habitat effects appeared to coincide with fish biomass at large scales and constrain the detection of reserve benefits. Until recently, coral habitats on northern reefs were likely to be healthier [35] and support greater fish biomasses than those in the south owing to a lower stress exposure such as COTS outbreaks, cyclones and bleaching [34, 57]. Marine reserves cannot escape from such environmental impacts. For example, in the Keppel Islands (central GBR), a decline in the density of the coral trout in both fished and protected areas was recorded after an extreme coral bleaching event [28]. Therefore, lower fish biomass on the southern reefs near Cooktown could reflect greater losses in habitat structure (i.e. coral cover) which may mask or limit the positive impact of marine reserves on fish stocks. The lower fish population size in the south could also reduce stock resilience through greater recruitment limitation. This would be expected to amplify the demographic significance of post-settlement mortality and therefore the ‘confounding’ effects of differences in habitat quality [80].

Low population size of highly-targeted species in the south could also suggest that fishing is likely to increase in a northward direction, as more commercial and recreational fishers move away from the most heavily used and exploited reef areas [50]. This could also explain why we observed fishing effects on the inshore north reefs but not on the outer north reefs, supporting the idea that fishing is likely to be greater in the more accessible nearshore (<50km) reefs than offshore (>100km) [50]. Low fish population size in offshore reefs compared to inshore reefs could also explain the lack of zoning effects offshore. On the GBR, inshore reefs tend to support higher abundance of both lutjanids and lethrinids compared to offshore reefs [81, 82]. Newman and Williams [81] showed that the cross-shelf variation in fish abundance on the central GBR was essentially due to highly fished species (lutjanid species *L*. *carponotatus* and *L*. *russeli)* that were characteristic of inshore and mid-shelf shallow reefs, but naturally rare on outer reefs [81]. Similarly, and in accordance with our results, Emslie et al. [82] observed that the abundance of *L*. *vitta* and *L*. *carponotatus* were highest on inshore reefs while that of *P*. *laevis* and *P*. *leopardus* occurred mainly on mid and outer-shelf reefs [82]. Here, low densities of highly targeted species were observed in offshore reefs (1.07 ± 0.1 ind. 120 m^2^) compared to inshore reefs (11.6 ± 1.7 ind. 120 m^2^). Therefore, the decline in predatory fish abundance from inshore to offshore reefs could have limited detectability of fishing effects in outer reefs.

Our results on non-targeted prey species contrast with those of Boaden and Kingsford [41] which examined coral trout on the ribbon reefs of the GBR. In their study, coral trout abundance was significantly depleted in fished reefs compared to reserves and benthic cover, which was indistinctive among management zones, was a poor predictor of prey density (mainly pomacentrids). Therefore, strong negative relationships were detected between predator density and that of small prey [41]. Here, no differences in the biomass of coral trout were detected on the southern outer reefs (which include the ribbon reefs), therefore no top-down effects were expected. However, on inshore reefs where a positive reserve effect on predators was detected, the apparent top-down effect on prey density was actually explained by variability in habitat characteristics between reserves and fished reefs. Here, habitat was a strong predictor of prey abundance limiting detectability of top-down effects. Strong effects of benthic habitat have also been found on other attributes of the fish assemblage that are susceptible to the impact of fishing such as mean and maximum size, functional diversity, and the proportion of herbivores among others [83]. This emphasizes how important it is to factor out the structuring influence of habitat on coral reef fish from the effect of management, and underlines the need of incorporating habitat structure into the design of surveys assessing the benefits of marine reserves including the ability to elicit trophic cascades on coral reefs (e.g. [1, 39, 40, 48, 83]). Differences in species composition of both predators and prey could also contribute to the contrasting results between studies. First, our analyses on prey included all fish species smaller than 10 cm while Boaden and Kingsford [41] separated prey by functional groups. Moreover, in the study of Boaden and Kingsford [41], coral trout and *Lutjanus carponotatus* were the strongest predictors of prey abundance, particularly omnivorous pomacentrids. In our study, coral trout biomass was indistinctive between inshore reserves and fished sites. However, while we detected that the density of *L*. *carponotatus* almost doubled in inshore reserves, it did not negatively correlate with the density of small omnivorous pomacentrids. The very low abundance of *L*. *carponotatus* detected here (3.1 ± 0.3 ind. 120 m^2^) could have limited detectability of top-down effects. Lack of detection of top-down effects in our study could also result from the variability among observers which accounted for more than 50% of the variability in prey abundance between zones. Our results show that the effect of surveyors was greater for small fish than for large mesopredatory fishes. Variation in fish counts or the detectability of small fish can result from poor environmental conditions (e.g. visibility), the surveying technique, the surveyor’s expertise and their ability to detect species with certain characteristics (e.g. small size, shyness, crypticity) [84, 85]. While observer effects are commonplace in fish studies, they were included explicitly in all relevant analyses.

This study adds to the growing evidence of the effect of reserve size on nearby fishing grounds [18, 20, 23, 24, 86]. The biomass of highly targeted species in fished sites declined near reserve boundaries and even more strongly when reserves were large. Because similar correlations were absent for less-targeted and non-targeted fish, it is likely that such patterns reflected fishing rather than habitat artefacts. Thus, it seems likely that fishers ‘fish the line’ [58, 87] and obtain greater benefits by targeting larger reserves. While a direct analysis of ‘catch per unit effort’ is needed to corroborate spillover effects, evidence of the migration of adult piscivores and larval export exist: 1) coral trout move distances of up to 7.5 km [88, 89] which is greater than the average distance between fished reefs and reserves estimated here, and 2) species such as the coral trout *P*. *maculatus* and the stripey snapper *L*. *carponotatus* have been found to export 83% and 55% of their offspring respectively to fished reefs within 30 km on the GBR [14].

While no baseline data on fish stocks exist for a temporal assessment of the effectiveness of marine reserves in the Far-Northern GBR, this study highlights the positive impact of marine reserves in the remote areas of the GBR. Moreover, it shows that the impact of spatial management is likely to be highly contextual and that comparing the efficiency of reserves must account for differences in habitat, among other attributes. In this case, we also needed to account for the counterintuitive effect of reserve size (i.e. decreasing fish biomass in nearby fishing sites) to detect potential spillover effects, and account for differences in spatial fishing effort and levels of compliance that are likely to counteract the magnitude of the reserve effect.

While this study lacks temporal replication and fluctuations in fish biomass due to life history or ontogenetic migrations may have also contributed to the lack of detection of management effects [89, 90], our results on southern reefs are similar to those observed by Casey et al. [42] for the same reefs in a different year and season. In addition, our study could only investigate two fished reefs inshore and only one on central-outer reefs. Therefore, our results need to be interpreted with a degree of caution and further sampling would be desirable. However, our results reinforce the need to distinguish the effects of changes in habitat structure from the effects of management when assessing the impact of marine reserves on coral reefs [46, 48, 83].

## Supporting information

S1 Fig Principle Coordinate Analysis (PCO) of targeted fish biomass. The observed variability in targeted fish biomass was unrelated to differences in habitat type among sites.
(TIF)

S2 Fig Principle Coordinate Analysis (PCO) of non-targeted fish biomass. The observed within-reef variability (PCO2) in the biomass structure of non-targeted fish was unrelated to environmental variables. Only Depth show a moderately strong correlation (Spearman = 0.5).
(TIF)

S3 Fig Fish biomass estimates per observer. Biomass of highly targeted species (mean ± SE) estimated per observer at each geographic location in fished (blue) and reserve sites (orange).
(TIF)

S1 Table Details of sampling design. Total number of benthic and fish transects performed at each reef site.
(DOCX)

S2 Table Fish species categorized by its relevance to commercial fisheries and main diet. Only large-bodied (TL > 30 cm) teleost are listed. Fishing categories based on the annual status report of Queensland commercial fisheries.
(DOCX)

S3 Table Site-level data used for testing zoning effects. Fish biomass per fishing category, percentage cover of principal benthic components, and other reef site attributes.
(XLS)

S4 Table Pair-wise test for significant interaction terms of zoning with geographic locations. Based on PERMANOVA results on benthic community structure and fish assemblage structure of targeted species.
(DOCX)

S5 Table Coral taxa categorized by main growth form. Relative contribution of coral genera (% cover) to the main growth forms identified in PCO and SIMPER analyses as major contributors to benthic community structure.
(DOCX)

S6 Table Pair-wise test for differences among zones at each geographic location. Based on PERMANOVA results on benthic community structure and fish assemblage structure of targeted species.
(DOCX)

S7 Table Results of linear mixed models testing zoning and habitat effects on fish biomass. Significant (p≤0.05) effects are shown in bold.
(DOCX)

S8 Table Variance components analysis of random effects. Estimates of the % variance of random effects included in the linear mixed models testing zoning effects on fish biomass.
(DOCX)
