# Supplementary figures and images for: Detecting conservation benefits of marine reserves on remote reefs of the northern GBR
Source: PLoS One. 2017 Nov 8;12(11):e0186146. doi: 10.1371/journal.pone.0186146 (PMC5695593; doi:10.1371/journal.pone.0186146)

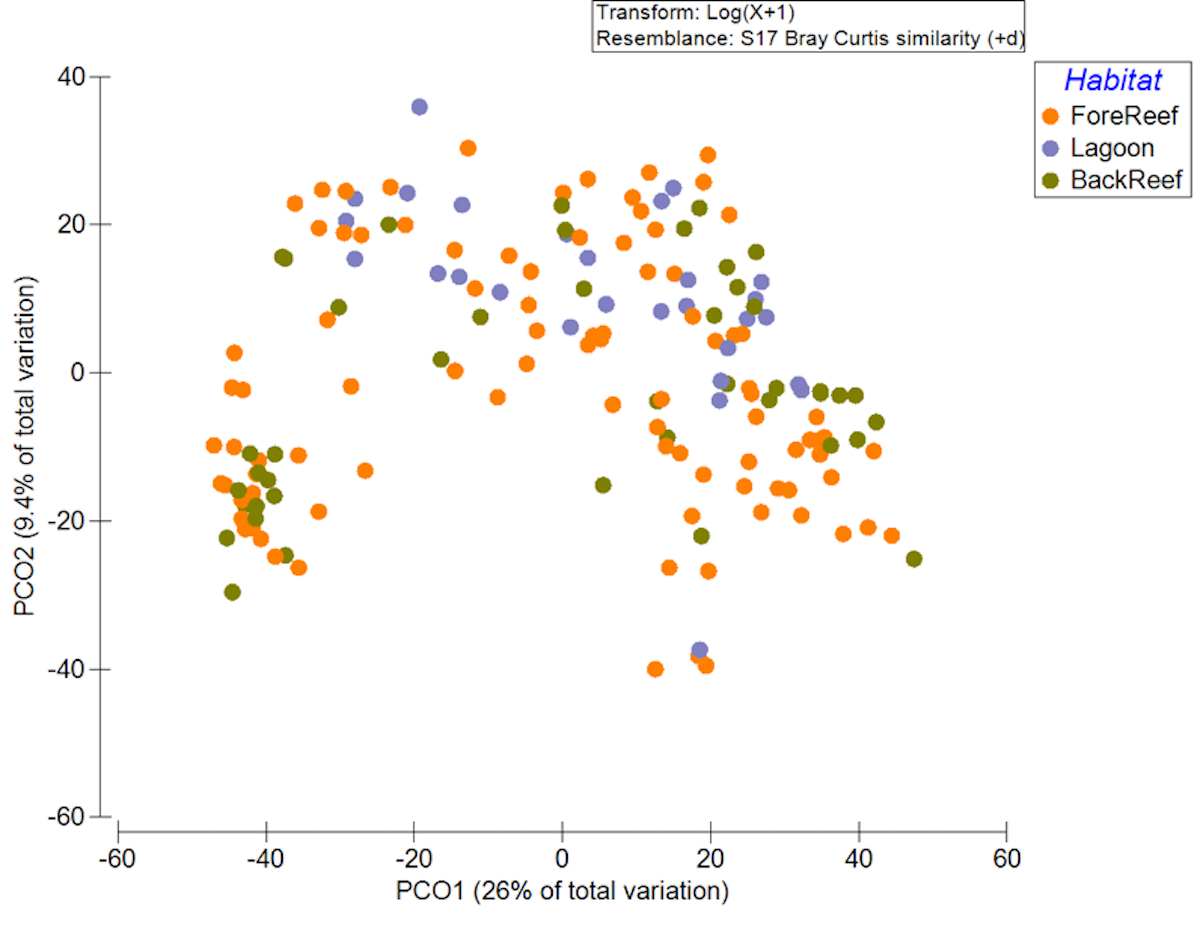

Supplement: S1 Fig — The observed variability in targeted fish biomass was unrelated to differences in habitat type among sites. (TIF) [file pone.0186146.s001.tif]

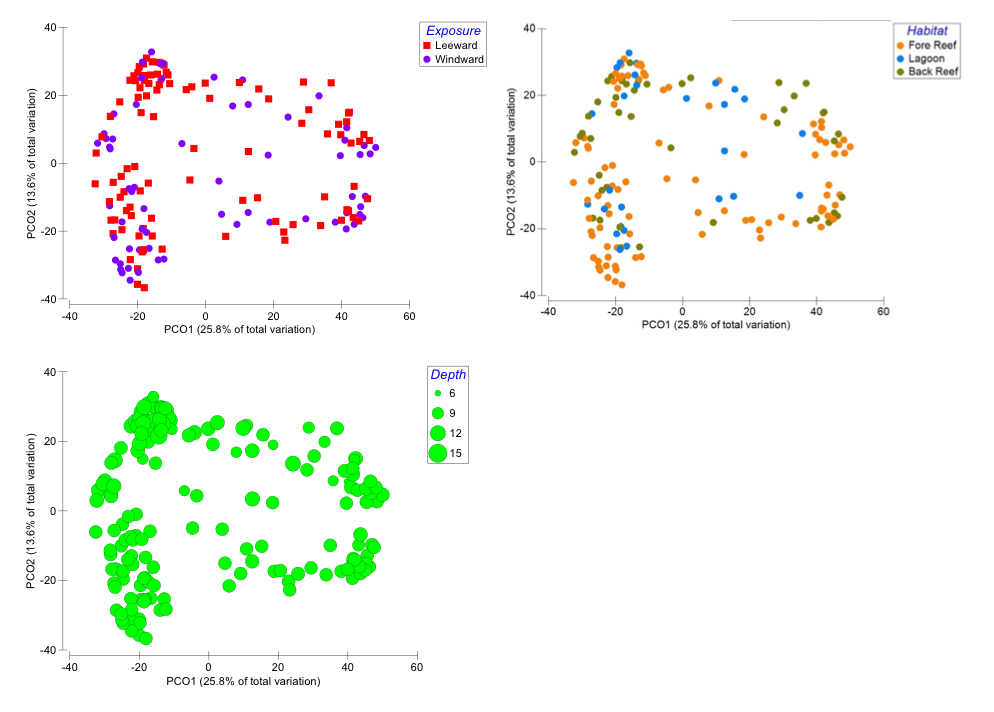

Supplement: S2 Fig — The observed within-reef variability (PCO2) in the biomass structure of non-targeted fish was unrelated to environmental variables. Only Depth show a moderately strong correlation (Spearman = 0.5). (TIF) [file pone.0186146.s002.tif]

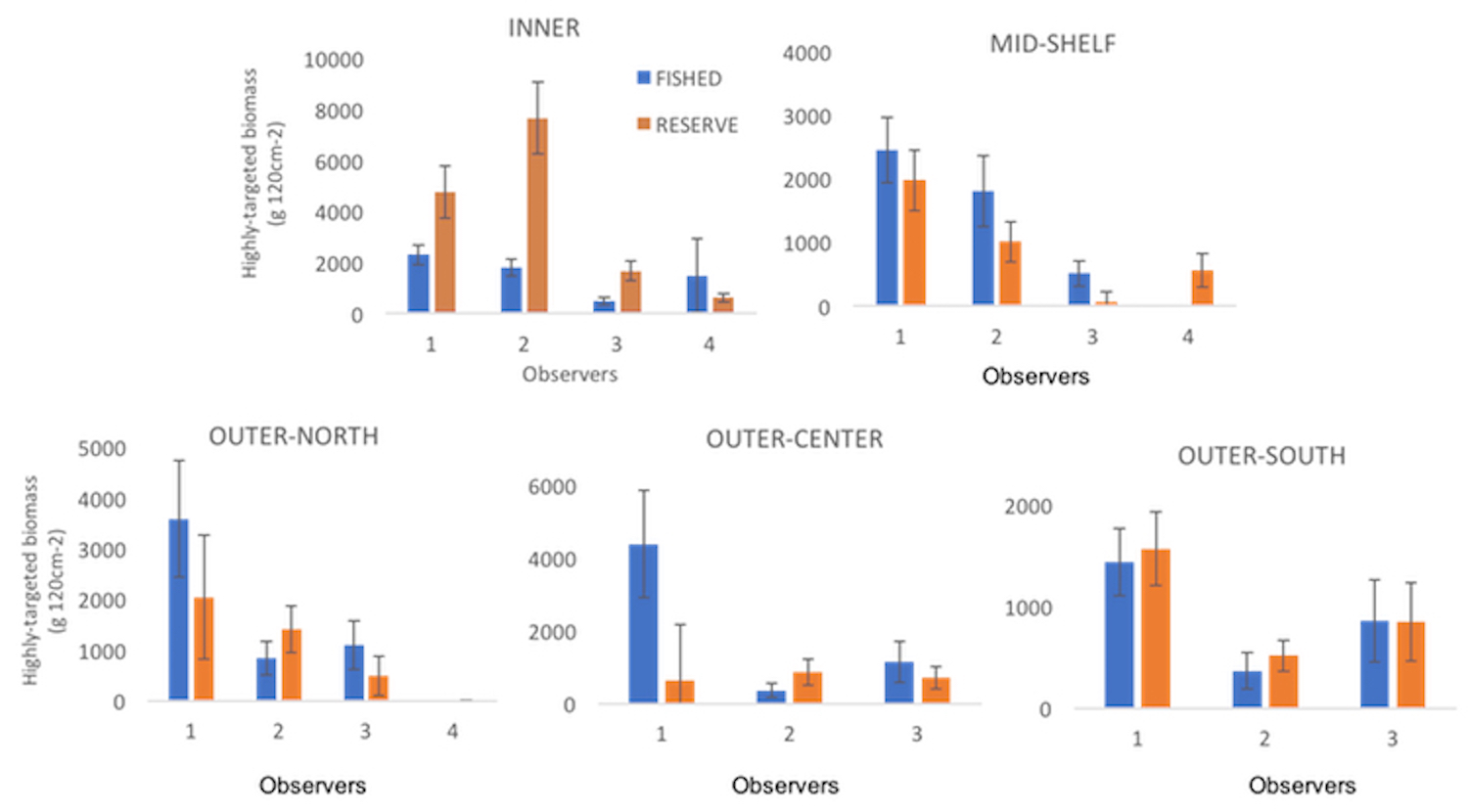

Supplement: S3 Fig — Biomass of highly targeted species (mean ± SE) estimated per observer at each geographic location in fished (blue) and reserve sites (orange). (TIF) [file pone.0186146.s003.tif]
