# Supplementary material for: Detecting conservation benefits of marine reserves on remote reefs of the northern GBR
Source: PLoS One. 2017 Nov 8;12(11):e0186146. doi: 10.1371/journal.pone.0186146 (PMC5695593; doi:10.1371/journal.pone.0186146)
Supplement: S1 Table — Total number of benthic and fish transects performed at each reef site. (DOCX) [file pone.0186146.s004.docx]

**S1 Table. Details of sampling design.** Total number of benthic and fish transects performed at each reef site

| Reef | Code | Zone | Subregion | No. Sites | No. LIT transects | No. photo transects | Total benthic transects | Total fish Transects |
| --- | --- | --- | --- | --- | --- | --- | --- | --- |
| Carter Reef | AUCT | Reserve | Outer-South | 7 | 9 | 1 | 10 | 23 |
| Eyrie Reef | AUEY | Reserve | Midshelf | 2 | 12 | 2 | 14 | 6 |
| Five Reefs | AU5R | Fished | Outer-North | 4 | 16 | 0 | 16 | 13 |
| Hicks Reef | AUHK | Fished | Outer-South | 6 | 8 | 3 | 11 | 27 |
| Hilder Reef | AUHI | Reserve | Outer-South | 6 | 28 | 2 | 30 | 28 |
| Jewell Reef | AUJW | Fished | Outer-South | 4 | 9 | 1 | 10 | 17 |
| Lark Reef | AULK | Reserve | Midshelf | 6 | 9 | 0 | 9 | 23 |
| Milman | AUMM | Reserve | Inner | 6 | 32 | 2 | 34 | 34 |
| Monkman | AUMO | Reserve | Inner | 6 | 26 | 4 | 30 | 35 |
| Parke | AUPK | Fished | Outer-South | 2 | 2 | 0 | 2 | 9 |
| Parkinson | AUPS | Reserve | Inner | 6 | 20 | 2 | 22 | 21 |
| Pearson | AUPE | Reserve | Midshelf | 6 | 26 | 2 | 28 | 28 |
| Ribbon_5 | AUR5 | Fished | Outer-South | 6 | 11 | 2 | 13 | 23 |
| Ribbon_6 | AUR6 | Reserve | Outer-South | 6 | 5 | 2 | 7 | 20 |
| Ribbon_7 | AUR7 | Reserve | Outer-South | 6 | 7 | 4 | 11 | 21 |
| Siddons | AUSD | Reserve | Midshelf | 6 | 36 | 3 | 39 | 32 |
| U/N (11-034) | AU34 | Reserve | Inner | 6 | 30 | 2 | 32 | 31 |
| U/N (11-096) | AU96 | Reserve | Outer-North | 6 | 28 | 3 | 31 | 17 |
| U/N (11-122) | AU122 | Fished | Outer-North | 4 | 19 | 1 | 20 | 18 |
| U/N (11-238) | AU238 | Fished | Outer-North | 4 | 21 | 2 | 23 | 15 |
| U/N (15-043) | AU43 | Fished | Midshelf | 6 | 15 | 2 | 17 | 25 |
| U/N(11-025) | AU25 | Fished | Inner | 6 | 29 | 3 | 32 | 31 |
| U/N(11-039) | AU39 | Fished | Inner | 6 | 28 | 1 | 29 | 27 |
| U/N(11-091) | AU91 | Reserve | Outer-North | 6 | 34 | 3 | 37 | 19 |
| U/N(13-061a) | AU61A | Reserve | Outer-Center | 6 | 17 | 2 | 19 | 27 |
| U/N(13-074) | AU74 | Reserve | Outer-Center | 6 | 35 | 2 | 37 | 22 |
| U/N(13-116) | AU116 | Fished | Outer-Center | 6 | 24 | 1 | 25 | 27 |
| Williamson | AUWL | Reserve | Midshelf | 6 | 7 | 3 | 10 | 22 |
| Wood | AUWD | Reserve | Outer-North | 4 | 17 | 2 | 19 | 19 |
| Yonge | AUYG | Reserve | Outer-South | 6 | 11 | 3 | 14 | 25 |
