# Supplementary material for: Detecting conservation benefits of marine reserves on remote reefs of the northern GBR
Source: PLoS One. 2017 Nov 8;12(11):e0186146. doi: 10.1371/journal.pone.0186146 (PMC5695593; doi:10.1371/journal.pone.0186146)
Supplement: S2 Table — Only large-bodied (TL > 30 cm) teleost are listed. Fishing categories based on the annual status report of Queensland commercial fisheries. (DOCX) [file pone.0186146.s005.docx]

**S2 Table. Fish species categorized by its relevance to commercial fisheries and main diet.** Only large-bodied (TL > 30 cm) teleost are listed. Fishing categories based on the annual status report of Queensland commercial fisheries.

| **Fishing value for Commercial fisheries** | **Family** | **Species** | **Main diet** |
| --- | --- | --- | --- |
| Highly fished | Lethrinidae | Lethrinus erythropterus | Piscivore |
|  | Lethrinidae | Lethrinus miniatus | Piscivore-Invertivore |
|  | Lethrinidae | Lethrinus nebulosus | Piscivore-Invertivore |
|  | Lutjanidae | Lutjanus adetii | Piscivore-Invertivore |
|  | Lutjanidae | Lutjanus carponotatus | Piscivore-Invertivore |
|  | Lutjanidae | Lutjanus russelli | Piscivore |
|  | Lutjanidae | Lutjanus sebae | Piscivore |
|  | Lutjanidae | Lutjanus vitta | Piscivore |
|  | Serranidae | Plectropomus areolatus | Piscivore |
|  | Serranidae | Plectropomus laevis | Piscivore |
|  | Serranidae | Plectropomus leopardus | Piscivore |
|  | Serranidae | Plectropomus maculatus | Piscivore-Invertivore |
|  | Serranidae | Plectropomus oligocanthus | Piscivore-Invertivore |
|  | Scombridae | Scomberomorus commerson | Piscivore |
|  | Serranidae | Variola albimarginata | Piscivore |
|  | Serranidae | Variola louti | Piscivore |
|  |  |  |  |
| Less fished | Carangidae | Scomberoides lysan | Piscivore |
|  | Carangidae | Seriola dumerili | Piscivore |
|  | Carangidae | Trachinotus blochii | Piscivore-Invertivore |
|  | Carangidae | Carangoides ferdau | Piscivore |
|  | Carangidae | Carangoides fulvoguttatus | Piscivore |
|  | Carangidae | Carangoides plagiotaenia | Piscivore |
|  | Carangidae | Caranx ignobilis | Piscivore |
|  | Carangidae | Caranx melampygus | Piscivore |
|  | Carangidae | Caranx papuensis | Piscivore |
|  | Carangidae | Caranx sexfasciatus | Piscivore |
|  | Labridae | Choerodon anchorago | Piscivore-Invertivore |
|  | Labridae | Choerodon cyanodus | Macroinvertivore |
|  | Labridae | Choerodon fasciatus | Piscivore-Invertivore |
|  | Labridae | Choerodon graphicus | Macroinvertivore |
|  | Labridae | Choerodon schoenleinii | Macroinvertivore |
|  | Lethrinidae | Gnathodentex aureolineatus | Piscivore-Invertivore |
|  | Lethrinidae | Gymnocranius grandoculis | Piscivore-Invertivore |
|  | Lethrinidae | Gymnocranius microdon | Macroinvertivore |
|  | Lethrinidae | Gymnocranius_sp | Piscivore-Invertivore |
|  | Lethrinidae | Lethrinus atkinsoni | Piscivore-Invertivore |
|  | Lethrinidae | Lethrinus erythracanthus | Piscivore |
|  | Lethrinidae | Lethrinus harak | Piscivore |
|  | Lethrinidae | Lethrinus laticaudis | Piscivore-Invertivore |
|  | Lethrinidae | Lethrinus lentjan | Piscivore-Invertivore |
|  | Lethrinidae | Lethrinus microdon | Piscivore |
|  | Lethrinidae | Lethrinus obsoletus | Macroinvertivore |
|  | Lethrinidae | Lethrinus olivaceus | Piscivore |
|  | Lethrinidae | Lethrinus rubrioperculatus | Piscivore |
|  | Lethrinidae | Lethrinus semicinctus | Piscivore-Invertivore |
|  | Lethrinidae | Lethrinus xanthochilus | Piscivore |
|  | Lethrinidae | Monotaxis grandoculis | Macroinvertivore |
|  | Lutjanidae | Aprion virescens | Piscivore |
|  | Lutjanidae | Lutjanus argentimaculatus | Piscivore-Invertivore |
|  | Lutjanidae | Lutjanus ehrenbergii | Piscivore |
|  | Lutjanidae | Lutjanus fulviflamma | Piscivore |
|  | Lutjanidae | Lutjanus fulvus | Piscivore-Invertivore |
|  | Lutjanidae | Aphareus furca | Piscivore |
|  | Lutjanidae | Lutjanus kasmira | Piscivore-Invertivore |
|  | Lutjanidae | Lutjanus lunulatus | Piscivore |
|  | Lutjanidae | Lutjanus lutjanus | Piscivore-Invertivore |
|  | Lutjanidae | Lutjanus monostigma | Piscivore |
|  | Lutjanidae | Lutjanus novemfasciatus | Piscivore |
|  | Lutjanidae | Lutjanus quinquelineatus | Piscivore-Invertivore |
|  | Lutjanidae | Lutjanus rivulatus | Piscivore |
|  | Lutjanidae | Lutjanus semicinctus | Piscivore |
|  | Lutjanidae | Lutjanus_sp | Piscivore |
|  | Lutjanidae | Symphorichthys spilurus | Piscivore |
|  | Serranidae | Aethaloperca rogaa | Piscivore |
|  | Serranidae | Anyperodon leucogrammicus | Piscivore |
|  | Serranidae | Cephalopholis argus | Piscivore |
|  | Serranidae | Cephalopholis boenak | Piscivore |
|  | Serranidae | Cephalopholis cyanostigma | Piscivore |
|  | Serranidae | Cephalopholis miniata | Piscivore |
|  | Serranidae | Cephalopholis sexmaculata | Piscivore |
|  | Serranidae | Cephalopholis spiloparaea | Piscivore-Invertivore |
|  | Serranidae | Epinephelus caeruleopunctatus | Piscivore |
|  | Serranidae | Epinephelus corallicola | Piscivore |
|  | Serranidae | Epinephelus cyanopodus | Piscivore |
|  | Serranidae | Epinephelus fasciatus | Piscivore-Invertivore |
|  | Serranidae | Epinephelus fuscoguttatus | Piscivore-Invertivore |
|  | Serranidae | Epinephelus howlandi | Piscivore |
|  | Serranidae | Epinephelus macrospilos | Piscivore |
|  | Serranidae | Epinephelus maculatus | Piscivore |
|  | Serranidae | Epinephelus malabaricus | Piscivore |
|  | Serranidae | Epinephelus merra | Piscivore |
|  | Serranidae | Epinephelus ongus | Piscivore-Invertivore |
|  | Serranidae | Epinephelus polyphekadion | Piscivore |
|  | Serranidae | Epinephelus quoyanus | Piscivore-Invertivore |
|  | Serranidae | Epinephelus socialis | Piscivore |
|  | Serranidae | Epinephelus tauvina | Piscivore |
|  | Serranidae | Epinephelus_sp | Piscivore |
|  | Serranidae | Psammoperca waigiensis | Piscivore-Invertivore |
|  |  |  |  |
| Not fished | Aulostomidae | Aulostomus chinensis | Piscivore-Invertivore |
|  | Balistidae | Balistoides viridescens | Macroinvertivore |
|  | Balistidae | Balistoides conspicillum | Macroinvertivore |
|  | Balistidae | Sufflamen chrysopterus | Macroinvertivore |
|  | Balistidae | Odonus niger | Microinvertivore |
|  | Balistidae | Pseudobalistes flavimarginatus | Macroinvertivore |
|  | Balistidae | Pseudobalistes fuscus | Macroinvertivore |
|  | Belonidae | Tylosurus crocodilus | Piscivore |
|  | Carangidae | Elagatis bipinnulata | Piscivore |
|  | Carangidae | Selar_sp | Macroinvertivore |
|  | Chaetodontidae | Chelmon rostratus | Microinvertivore |
|  | Chaetodontidae | Heniochus monoceros | Microinvertivore |
|  | Echeneidae | Echeneis naucrates | Piscivore |
|  | Fistulariidae | Fistularia commersonii | Piscivore |
|  | Haemulidae | Diagramma pictum | Piscivore-Invertivore |
|  | Haemulidae | Plectorhinchus albovittatus | Piscivore-Invertivore |
|  | Haemulidae | Plectorhinchus chaetodonoides | Piscivore-Invertivore |
|  | Haemulidae | Plectorhinchus chrysotaenia | Piscivore-Invertivore |
|  | Haemulidae | Plectorhinchus gibbosus | Piscivore-Invertivore |
|  | Haemulidae | Plectorhinchus lessonii | Piscivore-Invertivore |
|  | Haemulidae | Plectorhinchus lineatus | Piscivore-Invertivore |
|  | Haemulidae | Plectorhinchus multivittatum | Piscivore-Invertivore |
|  | Haemulidae | Plectorhinchus picus | Piscivore-Invertivore |
|  | Haemulidae | Plectorhinchus vittatus | Piscivore-Invertivore |
|  | Haemulidae | Plectorhinchus_sp | Piscivore-Invertivore |
|  | Holocentridae | Neoniphon opercularis | Macroinvertivore |
|  | Holocentridae | Sargocentron spiniferum | Piscivore-Invertivore |
|  | Holocentridae | Sargocentron tiere | Piscivore-Invertivore |
|  | Labridae | Anampses caeruleopunctatus | Macroinvertivore |
|  | Labridae | Bodianus loxozonus | Piscivore-Invertivore |
|  | Labridae | Cheilinus chlorourus | Macroinvertivore |
|  | Labridae | Cheilinus fasciatus | Macroinvertivore |
|  | Labridae | Cheilinus trilobatus | Piscivore-Invertivore |
|  | Labridae | Coris aygula | Macroinvertivore |
|  | Labridae | Coris bulbifrons | Macroinvertivore |
|  | Labridae | Coris dorsomacula | Macroinvertivore |
|  | Labridae | Coris gaimard | Macroinvertivore |
|  | Labridae | Epibulus insidiator | Piscivore-Invertivore |
|  | Labridae | Hemigymnus melapterus | Macroinvertivore |
|  | Labridae | Hologymnosus doliatus | Piscivore-Invertivore |
|  | Labridae | Oxycheilinus digramma | Macroinvertivore |
|  | Labridae | Oxycheilinus unifasciatus | Piscivore |
|  | Mullidae | Mulloidichthys flavolineatus | Macroinvertivore |
|  | Mullidae | Mulloidichthys vanicolensis | Macroinvertivore |
|  | Mullidae | Parupeneus barberinus | Macroinvertivore |
|  | Mullidae | Parupeneus bifasciatus | Piscivore-Invertivore |
|  | Mullidae | Parupeneus ciliatus | Macroinvertivore |
|  | Mullidae | Parupeneus crassilabris | Macroinvertivore |
|  | Mullidae | Parupeneus cyclostomus | Piscivore-Invertivore |
|  | Mullidae | Parupeneus indicus | Piscivore-Invertivore |
|  | Mullidae | Parupeneus multifasciatus | Macroinvertivore |
|  | Mullidae | Parupeneus pleurostigma | Piscivore-Invertivore |
|  | Mullidae | Parupeneus spilurus | Macroinvertivore |
|  | Muraenidae | Gymnothorax javanicus | Piscivore |
|  | Nemipteridae | Scolopsis monogramma | Piscivore-Invertivore |
|  | Nemipteridae | Pentapodus caninus | Piscivore-Invertivore |
|  | Pomacanthidae | Pomacanthus semicirculatus | Microinvertivore |
|  | Pomacanthidae | Pomacanthus sexstriatus | Microinvertivore |
|  | Priacanthidae | Priacanthus blochii | Piscivore-Invertivore |
|  | Priacanthidae | Priacanthus hamrur | Piscivore-Invertivore |
|  | Scombridae | Gymnosarda unicolor | Piscivore |
|  | Scombridae | Sarda orientalis | Piscivore |
|  | Scombridae | Thunnus obesus | Piscivore |
|  | Scorpaenidae | Pterois volitans | Piscivore |
|  | Sphyraenidae | Sphyraena flavicauda | Piscivore |
|  | Sphyraenidae | Sphyraena jello | Piscivore |
|  | Sphyraenidae | Sphyraena qenie | Piscivore |
|  | Synodontidae | Synodus variegatus | Piscivore |
|  | Tetraodontidae | Arothron stellatus | Macroinvertivore |
|  | Tetraodontidae | Arothron hispidus | Macroinvertivore |
