# Supplementary material for: Detecting conservation benefits of marine reserves on remote reefs of the northern GBR
Source: PLoS One. 2017 Nov 8;12(11):e0186146. doi: 10.1371/journal.pone.0186146 (PMC5695593; doi:10.1371/journal.pone.0186146)
Supplement: S4 Table — Based on PERMANOVA results on benthic community structure and fish assemblage structure of targeted species. (DOCX) [file pone.0186146.s007.docx]

**S4 Table. Pair-wise test for significant interaction terms of zoning with geographic locations.** Based on PERMANOVA results on benthic community structure and fish assemblage structure of targeted species.

| **Benthic community structure** | | | | | | | |
| --- | --- | --- | --- | --- | --- | --- | --- |
|  |  |  | Zone*Shelf position | |  |  | |
|  | **Inner vs Mid-shelf** | | **Outer-Mid-Shelf** | | **Outer vs Inner** | | |
| **Zoning** | t | *P* adj | t | p | t | *P* adj | |
| fished | 1.9 | **0.004** | 4.61 | **0.002** | 4.41 | **< 0.001** | |
| no-take | 3.27 | **0.005** | 2.73 | **0.001** | 4.3 | **< 0.001** | |
| no-entry | 2.01 | **0.003** | 3.1 | **0.001** | 4.31 | **< 0.001** | |
|  |  |  | Zone* Sub-region | |  |  | |
|  | **central vs north** | | **central vs south** | | **north vs south** | | |
| **Zoning** | t | *P* adj | t | p | t | *P* adj | |
| fished | 1.39 | 0.08 | 1.63 | **0.04** | 3 | **0.001** | |
| no-take | 3.2 | **0.005** | 1.9 | **0.006** | 1.74 | **0.01** | |
| no-entry | 2.47 | **0.003** | 1.74 | **0.02** | 1.52 | **0.04** | |
| Zone*Exposure (Exp) | | |  | Shelf position*Exp | | | |
| **Leeward vs Windward** | | |  | **Leeward vs Windward** | | | |
| **Zoning** | t | *P* adj |  | **Shelf position** | t | *P* adj | |
| fished | 0.85 | 0.62 |  | Inner | 1.59 | 0.06 | |
| no-take | 1.66 | **0.03** |  | Mid-shelf | 1.33 | 0.13 | |
| no-entry | 0.88 | 0.91 |  | Outer | 1.35 | 0.09 | |
|  | | | | | | | |
|  | **Inner vs Mid-shelf** | | **Outer-Mid-Shelf** | | **Outer vs Inner** | | |
| **Exposure** | t | *P* adj | t | *P* adj | t | *P* adj | |
| Leeward | 2.25 | **0.002** | 4.26 | **< 0.001** | 4.7 | **< 0.001** | |
| Windward | 2.33 | **0.001** | 3.25 | **< 0.001** | 5.08 | **< 0.001** | |
|  | | | | | | |  |
| **Fish assemblage structure (fished species)** | | | | | | |  |
|  |  |  | Zone*Shelf position | |  |  | |
| Shelf position | **Inner vs Mid-shelf** | | **Outer-Mid-Shelf** | | **Outer vs Inner** | | |
| **Zoning** | t | *P* adj | t | *P* adj | t | *P* adj | |
| fished | 1.00 | 0.45 | 3.34 | **0.001** | 2.95 | **< 0.001** | |
| no-take | 2.09 | **0.004** | 2.75 | **< 0.001** | 3.29 | **< 0.001** | |
| no-entry | 2.65 | **0.002** | 2.61 | **< 0.001** | 3.68 | **< 0.001** | |
|  |  |  | Zone*Sub-region | |  |  | |
| Sub-region | **central vs north** | | **central vs south** | | **north vs south** | | |
| **Zoning** | t | *P* adj | t | *P* adj | t | *P* adj | |
| fished | 1.85 | **0.002** | 1.29 | 0.08 | 1.56 | **0.001** | |
| no-take | 1.85 | **0.002** | 1.71 | **0.001** | 1.49 | 0.01 | |
| no-entry | 1.69 | **0.002** | 1.04 | 0.37 | 1.77 | **< 0.001** | |

*P* adj: Adjusted p-values using the Benjamini & Hochberg (BH) procedure
