# Supplementary material for: Detecting conservation benefits of marine reserves on remote reefs of the northern GBR
Source: PLoS One. 2017 Nov 8;12(11):e0186146. doi: 10.1371/journal.pone.0186146 (PMC5695593; doi:10.1371/journal.pone.0186146)
Supplement: S5 Table — Relative contribution of coral genera (% cover) to the main growth forms identified in PCO and SIMPER analyses as major contributors to benthic community structure. (DOCX) [file pone.0186146.s008.docx]

**S5 Table. Coral taxa categorized by main growth form.** Relative contribution of coral genera (% cover) to the main growth forms identified in PCO and SIMPER analyses as major contributors to benthic community structure.

| **Genera** | **Branching** | |  | **Genera** | **Massive** |  | **Genera** | **Plate** |
| --- | --- | --- | --- | --- | --- | --- | --- | --- |
| *Acropora* | | 47.4 |  | *Porites* | 72.5 |  | *Montipora* | 42.3 |
| *Pocillopora* | | 20.8 |  | *Goniopora* | 8.9 |  | *Echinopora* | 23.2 |
| *Stylophora* | | 10.9 |  | *Favia* | 7.0 |  | *Pachyseris* | 16.1 |
| *Seriatopora* | | 6.7 |  | *Goniastrea* | 5.0 |  | *Isopora* | 12.8 |
| *Millepora* | | 4.5 |  | *Diploastrea* | 4.8 |  | *Turbinaria* | 5.6 |
| *Isopora* | | 3.2 |  | *Montastrea* | 1.6 |  |  |  |
| *Porites* | | 2.4 |  |  |  |  |  |  |
| *Pachyseris* | | 1.7 |  |  |  |  |  |  |
| *Pavona* | | 1.2 |  |  |  |  |  |  |
| *Montipora* | | 0.9 |  |  |  |  |  |  |
