# Supplementary material for: Detecting conservation benefits of marine reserves on remote reefs of the northern GBR
Source: PLoS One. 2017 Nov 8;12(11):e0186146. doi: 10.1371/journal.pone.0186146 (PMC5695593; doi:10.1371/journal.pone.0186146)
Supplement: S6 Table — Based on PERMANOVA results on benthic community structure and fish assemblage structure of targeted species. (DOCX) [file pone.0186146.s009.docx]

**S6 Table.** **Pair-wise test for differences among zones at each geographic location.**  Based on PERMANOVA results on benthic community structure and fish assemblage structure of targeted species.

| **Benthic community structure** | | |  |  |  |  |
| --- | --- | --- | --- | --- | --- | --- |
|  |  |  |  |  |  |  |
|  | **Fished vs No take** | | **Fished vs No entry** | | **No entry vs No take** | |
| **Shelf position** | t | *P* adj | t | *P* adj | t | *P* adj |
| Inner | 0.87 | 0.61 | 1.34 | 0.13 | 1.14 | 0.28 |
| Mid-Shelf | 1.42 | 0.11 | 2.06 | **0.009** | 1.96 | **0.004** |
| Outer | 1.63 | 0.06 | 1.61 | **0.04** | 1.35 | 0.11 |
|  | **Fished vs No take** | | **Fished vs No entry** | | **No entry vs No take** | |
| **Sub-region** | t | *P* adj | t | *P* adj | t | *P* adj |
| North | 1.44 | 0.09 | 1.98 | **0.009** | 1.75 | **0.01** |
| Central | 1.92 | **0.01** | 1.27 | 0.20 | 1.79 | **0.02** |
| South | 1.13 | 0.29 | 1.46 | 0.09 | 0.98 | 0.44 |
| **Fish assemblage structure (highly fished and less fished species)** | | | | | | |
|  |  |  |  |  |  |  |
|  | **Fished vs No take** | | **Fished vs No entry** | | **No entry vs No take** | |
| **Shelf position** | t | *P* adj | t | *P* adj | t | *P* adj |
| Inner | 0.98 | 0.56 | 1.60 | **0.03** | 1.33 | 0.27 |
| Mid-Shelf | 1.72 | **0.005** | 1.59 | **0.004** | 0.84 | 0.80 |
| Outer | 1.09 | 0.40 | 1.12 | 0.43 | 0.82 | 0.79 |
|  | **Fished vs No take** | | **Fished vs No entry** | | **No entry vs No take** | |
| **Sub-region** | t | *P* adj | t | *P* adj | t | *P* adj |
| North | 1.38 | 0.09 | 1.60 | **0.04** | 1.31 | 0.18 |
| Central | 1.27 | 0.22 | 1.17 | 0.31 | 1.30 | 0.22 |
| South | 1.01 | 0.55 | 0.79 | 0.83 | 0.79 | 0.92 |
|  |  |  |  |  |  |  |

*P* adj: Adjusted p-values using the Benjamini & Hochberg (BH) procedure
