# Supplementary material for: Detecting conservation benefits of marine reserves on remote reefs of the northern GBR
Source: PLoS One. 2017 Nov 8;12(11):e0186146. doi: 10.1371/journal.pone.0186146 (PMC5695593; doi:10.1371/journal.pone.0186146)
Supplement: S7 Table — Significant (p≤0.05) effects are shown in bold. (DOCX) [file pone.0186146.s010.docx]

**S7 Table.** **Results of Linear mixed models testing zoning and habitat effects on fish biomass.** Significant (p≤0.05) effects are shown in bold.

| Location | Variable | Effect | Estimate | SE | df | t | p-value |
| --- | --- | --- | --- | --- | --- | --- | --- |
| Inner –north | Highly targeted | (Intercept) | 7.17 | 0.25 | 28.00 | 28.72 | <0.0001 |
|  | (log+1) | Reserve | 0.85 | 0.29 | 28.00 | 2.95 | **0.01** |
|  |  | Benthos_PCO2 | 0.01 | 0.01 | 28.00 | 0.80 | 0.43 |
|  |  | Benthos_PCO1 | 0.00 | 0.01 | 28.00 | -0.12 | 0.91 |
|  |  | Windward | 0.11 | 0.28 | 28.00 | 0.41 | 0.69 |
|  | Less targeted | (Intercept) | 7.26 | 0.61 | 5.91 | 11.93 | 0.00 |
|  | (log+1) | Reserve | 0.33 | 0.66 | 4.63 | 0.51 | 0.64 |
|  |  | Windward (W) | -0.57 | 0.49 | 24.70 | -1.18 | 0.25 |
|  |  | Benthos_PCO2 | -0.02 | 0.01 | 23.35 | -1.50 | 0.15 |
|  |  | Benthos_PCO1 | 0.01 | 0.01 | 25.62 | 1.08 | 0.29 |
|  |  | Reserve * W | 1.09 | 0.58 | 24.57 | 1.87 | 0.07 |
|  | Non-targeted | (Intercept) | 6.39 | 0.34 | 4.28 | 18.82 | <0.0001 |
|  | (log+1) | Reserve | -0.32 | 0.28 | 3.07 | -1.15 | 0.33 |
|  |  | Benthos_PCO2 | 0.01 | 0.01 | 22.49 | 2.36 | **0.03** |
|  |  | Benthos_PCO1 | 0.00 | 0.01 | 24.86 | -0.82 | 0.42 |
|  |  | Windward | 0.03 | 0.11 | 22.93 | 0.24 | 0.81 |
| MidShelf | Highly targeted | (Intercept) | 6.38 | 0.76 | 18.54 | 8.42 | <0.0001 |
| (north and south) | (log+1) | Reserve | 0.20 | 0.67 | 26.83 | 0.30 | 0.77 |
|  |  | Benthos_PCO2 | -0.05 | 0.03 | 25.58 | -1.95 | 0.06 |
|  |  | Benthos_PCO1 | 0.01 | 0.01 | 26.99 | 1.01 | 0.32 |
|  |  | Windward | 0.64 | 0.55 | 26.65 | 1.17 | 0.25 |
|  | Less targeted | (Intercept) | 4.61 | 0.76 | 9.03 | 6.06 | <0.0001 |
|  | (log+1) | Reserve | 0.95 | 0.60 | 25.17 | 1.59 | 0.12 |
|  |  | Benthos_PCO2 | -0.03 | 0.02 | 24.69 | -1.33 | 0.20 |
|  |  | Benthos_PCO1 | 0.03 | 0.01 | 24.04 | 2.01 | 0.06 |
|  |  | Windward | 0.41 | 0.50 | 26.80 | 0.80 | 0.43 |
|  | Non-targeted | (Intercept) | 6.26 | 0.55 | 3.75 | 11.43 | <0.001 |
|  | (log+1) | Reserve | 0.25 | 0.58 | 3.34 | 0.42 | 0.70 |
|  |  | Benthos_PCO1 | -0.02 | 0.01 | 21.62 | -3.32 | **0.003** |
|  |  | Benthos_PCO2 | 0.02 | 0.01 | 18.87 | 3.40 | **0.003** |
|  |  | Windward | -0.13 | 0.13 | 21.45 | -0.98 | 0.34 |
| Outer –north | Highly targeted | (Intercept) | 4.65 | 1.65 | 3.42 | 2.81 | 0.06 |
|  | (log+1) | Reserve | 0.22 | 0.87 | 23.74 | 0.25 | 0.80 |
|  |  | Benthos_PCO2 | -0.04 | 0.03 | 23.11 | -1.08 | 0.29 |
|  |  | Benthos_PCO1 | -0.05 | 0.03 | 23.22 | -1.95 | 0.06 |
|  |  | Windward | 0.02 | 0.63 | 23.14 | 0.04 | 0.97 |
|  | Less targeted | (Intercept) | 5.97 | 1.54 | 5.11 | 3.88 | 0.01 |
|  | (Sqrt-root) | Reserve | -0.15 | 1.07 | 7.50 | -0.14 | 0.89 |
|  |  | Benthos_PCO2 | 0.01 | 0.04 | 22.21 | 0.37 | 0.71 |
|  |  | Benthos_PCO1 | 0.02 | 0.03 | 23.15 | 0.57 | 0.58 |
|  |  | Windward | 0.96 | 0.67 | 21.85 | 1.42 | 0.17 |
|  | Non-targeted | (Intercept) | 7.06 | 0.20 | 7.78 | 34.55 | <0.0001 |
|  | (log+1) | Reserve | -0.22 | 0.21 | 26.00 | -1.01 | 0.32 |
|  |  | Benthos_PCO1 | 0.01 | 0.01 | 24.87 | 1.73 | 0.10 |
|  |  | Benthos_PCO2 | 0.00 | 0.01 | 25.35 | -0.23 | 0.82 |
|  |  | Windward | 0.23 | 0.16 | 25.20 | 1.41 | 0.17 |
| Outer –central | Highly targeted | (Intercept) | 7.60 | 1.38 | 1.49 | 5.49 | 0.06 |
|  | (log+1) | Reserve | 0.08 | 1.21 | 0.90 | 0.07 | 0.96 |
|  |  | Benthos_PCO2 | -0.01 | 0.04 | 11.91 | -0.28 | 0.79 |
|  |  | Benthos_PCO1 | 0.01 | 0.02 | 12.45 | 0.35 | 0.73 |
|  |  | Windward | -0.87 | 0.66 | 10.88 | -1.33 | 0.21 |
|  | Less targeted | (Intercept) | 6.36 | 0.49 | 9.10 | 13.03 | <0.0001 |
|  | (log+1) | Reserve | 1.60 | 0.64 | 12.99 | 2.51 | **0.03** |
|  |  | Benthos_PCO2 | 0.00 | 0.04 | 13.00 | 0.13 | 0.90 |
|  |  | Benthos_PCO1 | -0.01 | 0.02 | 9.87 | -0.48 | 0.64 |
|  |  | Windward | -1.26 | 0.67 | 12.36 | -1.88 | 0.08 |
|  | Non-targeted | (Intercept) | 6.94 | 0.44 | 1.07 | 15.64 | 0.03 |
|  | (log+1) | Reserve | 0.22 | 0.44 | 0.79 | 0.49 | 0.73 |
|  |  | Benthos_PCO2 | -0.02 | 0.02 | 10.80 | -1.20 | 0.26 |
|  |  | Benthos_PCO1 | 0.00 | 0.01 | 12.88 | 0.12 | 0.90 |
|  |  | Windward | -0.13 | 0.30 | 10.77 | -0.44 | 0.67 |
| Outer south | Highly targeted | (Intercept) | 7.03 | 1.16 | 7.07 | 6.08 | <0.001 |
|  | (log+1) | Reserve | -0.84 | 1.12 | 6.02 | -0.76 | 0.48 |
|  |  | Benthos_PCO2 | 0.00 | 0.03 | 42.86 | -0.01 | 1.00 |
|  |  | Benthos_PCO1 | 0.01 | 0.03 | 38.32 | 0.51 | 0.61 |
|  |  | Windward | -1.77 | 0.80 | 40.46 | -2.20 | **0.03** |
|  | Less targeted | (Intercept) | 7.06 | 0.54 | 3.99 | 13.00 | <0.001 |
|  | (log+1) | Reserve | 0.32 | 0.40 | 42.20 | 0.79 | 0.43 |
|  |  | Benthos_PCO2 | -0.02 | 0.02 | 42.88 | -0.99 | 0.33 |
|  |  | Benthos_PCO1 | 0.02 | 0.01 | 42.03 | 1.45 | 0.15 |
|  |  | Windward | -0.43 | 0.40 | 43.00 | -1.06 | 0.30 |
|  | Non-targeted | (Intercept) | 6.58 | 0.41 | 1.38 | 16.11 | 0.02 |
|  | (log+1) | Reserve | 0.21 | 0.16 | 42.03 | 1.33 | 0.19 |
|  |  | Benthos_PCO2 | 0.00 | 0.01 | 42.20 | 0.75 | 0.46 |
|  |  | Benthos_PCO1 | -0.01 | 0.00 | 42.01 | -1.05 | 0.30 |
|  |  | Windward | -0.04 | 0.16 | 42.29 | -0.24 | 0.81 |
