# Supplementary material for: Detecting conservation benefits of marine reserves on remote reefs of the northern GBR
Source: PLoS One. 2017 Nov 8;12(11):e0186146. doi: 10.1371/journal.pone.0186146 (PMC5695593; doi:10.1371/journal.pone.0186146)
Supplement: S8 Table — Estimates of the % variance of random effects included in the linear mixed models testing zoning effects on fish biomass. (DOCX) [file pone.0186146.s011.docx]

**S8 Table.** **Variance components analysis of random effects.** Estimates of the % variance of random effects included in the linear mixed models testing zoning effects on fish biomass.
